# Panobinostat potentiates adagrasib-induced cell death by triggering autophagy in human non-small cell lung cancer

**DOI:** 10.1038/s41420-025-02657-9

**Published:** 2025-08-01

**Authors:** Hui Lu, Wenying Fu, Yiqun Xia, Ying Yan, Chongchong Shu, Yinghua Chen, Chenxin Xu, Peisen Zheng, Xin Shen, Ri Cui, Peng Zou, Daoyong Ni

**Affiliations:** 1 Affiliated Yueqing Hospital, Wenzhou Medical University, Wenzhou, China; 2 School of Pharmaceutical Sciences, Wenzhou Medical University, Wenzhou, China; 3 The First Affiliated Hospital of Wenzhou Medical University, Wenzhou Medical University, Wenzhou, China

**Keywords:** Non-small-cell lung cancer, Cancer therapy

## Abstract

Adagrasib, a KRASG12C inhibitor, recently received accelerated approval from the US FDA for the treatment of patients diagnosed with KRASG12C-mutated non-small cell lung cancer. Although adagrasib has demonstrated excellent clinical efficacy and good safety, the molecular mechanism underlying the antitumor activity of adagrasib remains elusive. Here, we report that adagrasib treatment markedly inhibited the growth of cells harboring the KRASG12C mutation, whereas the non-KRASG12C cell lines H1299 and PC-9 were also sensitive to adagrasib, indicating that adagrasib exerted off-target effects. Mechanism studies indicated that adagrasib treatment reduced the level of NRF2 via upregulating its ubiquitination, and NRF2 overexpression can reverse the adagrasib-induced cell death in H23 and H1299 cells. Furthermore, adagrasib treatment significantly increased the cellular ROS level and thereby activating autophagy and AKT signaling pathways in H23 and H1299 cells. Importantly, combination of adagrasib with panobinostat demonstrated enhanced antitumor activity in vitro and in vivo. Overall, our data elucidate a novel mechanism of adagrasib, which will be critical for the clinical application of adagrasib.

## Introduction

Kirsten rat sarcoma viral oncogene homolog (KRAS) is among the most commonly mutated genes observed in various solid tumors [1–3]. KRAS mutations are found in approximately 25% individuals diagnosed with non-small cell lung cancer (NSCLC), who often have a less favorable outcome compared to those without these mutations [4]. Historically, KRAS has been viewed as a difficult target for drug development due to the absence of appropriate binding pockets. However, this situation has recently changed with the discovery of potent KRASG12C inhibitors. Among these, sotorasib and adagrasib have exhibited excellent antitumor activity and were recently approved by the U.S. FDA for treating individuals with KRASG12C-mutated NSCLC [4–6]. These approvals mark an important advancement in targeted therapy for this specific subset of lung cancer, providing new options for patients who previously had limited treatment alternatives.

Despite KRASG12C inhibitors sotorasib and adagrasib have shown excellent antitumor activity, they still face several important issues. One is drug resistance, the antitumor activity of sotorasib or adagrasib is restricted by both genetic and nongenetic resistance mechanisms [7–9]. For instance, the stimulation of SHP2 and PI3K/AKT pathways is involved in cell resistance to KRASG12C inhibitors, complicating treatment outcomes and limiting the effectiveness of these targeted therapies [10–12]. Another concern is the off-target effect and their underlying mechanism of these inhibitors. A recent study suggests that sotorasib can covalently modify the functional cysteine of KEAP1 and inhibit its enzymatic activity [13]. However, there is limited understanding of the off-target effects and their underlying mechanisms of adagrasib.

Panobinostat is a potent histone deacetylase (HDAC) inhibitor approved for the treatment of multiple myeloma and is under investigation for various hematologic and solid malignancies [14, 15]. By inhibiting histone deacetylases, panobinostat promotes the accumulation of acetylated histones and other proteins, leading to altered gene expression, cell cycle arrest, and cell death in cancer cells. Its broad-spectrum activity also affects non-histone substrates, influencing diverse cellular processes such as DNA damage repair, angiogenesis, and immune response [16, 17]. Due to its strong antitumor effects, panobinostat is often explored in combination with other therapeutic agents to enhance efficacy and overcome resistance. In NSCLC, panobinostat demonstrates potent antitumor activity and enhances tumor sensitivity to carboplatin [18]. Moreover, combining panobinostat with osimertinib synergistically reduces the viability of various osimertinib-resistant cell lines [19].

Here, we investigated the antitumor activity of adagrasib in NSCLC cells and observed that adagrasib treatment markedly inhibited the growth of H23, Calu-1, PC-9 and H1299 cells. Furthermore, we explored the underlying molecular mechanisms and demonstrated that NRF2 inhibition is pivotal for adagrasib-induced cell death. Importantly, we found that combining adagrasib with panobinostat elicits synthetic lethality in vivo. In summary, our study identifies a novel mechanism for adagrasib, providing a theoretical basis for its further clinical application. Additionally, our findings propose a new drug combination for the clinical management of lung cancer.

## Results

### The growth inhibitory effect of adagrasib on NSCLC cells

**Fig. 1:**
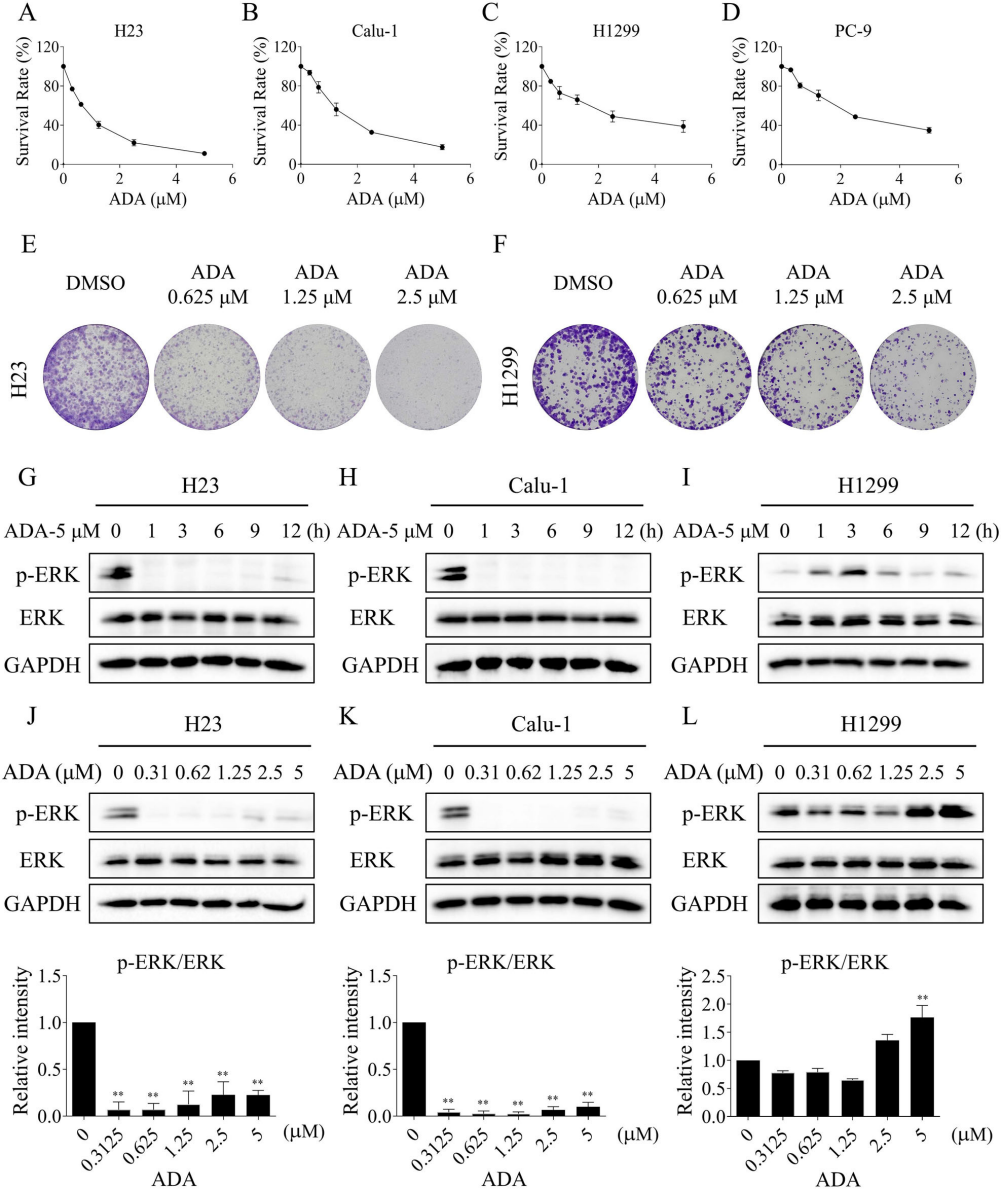
The growth inhibitory effect of ADA on NSCLC cells. **A**–**D** H23, Calu-1, H1299, and PC-9 cells were exposed to varying concentrations of adagrasib (ADA) for 24 h, after which cell viability was assessed. **E**, **F** H23 and H1299 cells were exposed to varying concentrations of ADA for 24 h. After this, the medium was replaced with fresh medium, and the cells were cultured for an additional 7 days before colony formation was assessed. **G**–**I** H23, Calu-1, and H1299 cells were treated with ADA (5 μM) for specified durations. The protein levels of p-ERK, ERK, and GAPDH were assessed by Western blot. **J**–**L** H23 and Calu-1 cells were treated with varying concentrations of ADA for 1 h, while H1299 cells were treated for 3 h. The protein levels of p-ERK, ERK, and GAPDH were assessed by Western blot. **p < 0.01, as determined by One-way ANOVA.

We first examined the growth-inhibitory effect of adagrasib (ADA) on human NSCLC cells. We set the maximum concentration of ADA to 5 μM based on the pharmacokinetic results in clinical patients [20]. Consistent with previous studies, ADA treatment significantly inhibited the growth of KRASG12C cell lines H23 and Calu-1, whereas the cell viability of H1299 and PC-9 (non-KRASG12C cell lines) was also inhibited by ADA in a dose-dependent manner (Fig. 1A–D). In addition, ADA can concentration-dependently inhibit the colony formation of H23 and H1299 (Fig. 1E, F). Western blotting results indicated that the phosphorylation of ERK was markedly inhibited by ADA in H23 and Calu-1 cells, but not in H1299 cells (Fig. 1G–I). Notably, the phosphorylation of ERK was significantly inhibited by ADA at all concentrations in H23 and Calu-1 cells. However, the modulation of p-ERK by ADA in H1299 cells was concentration-dependent. Specifically, we observed an inhibitory effect when the ADA concentration was below or equal to 1.25 μM. Conversely, an elevation of p-ERK is noted when the ADA concentration exceeds or equals 2.5 μM in H1299 cells (Fig. 1J–L). These results demonstrate that ADA exerts an off-target effect, the antitumor activity of ADA does not entirely depend on KRASG12C mutation.

### NRF2 inhibition is vital for ADA-induced cell death

**Fig. 2:**
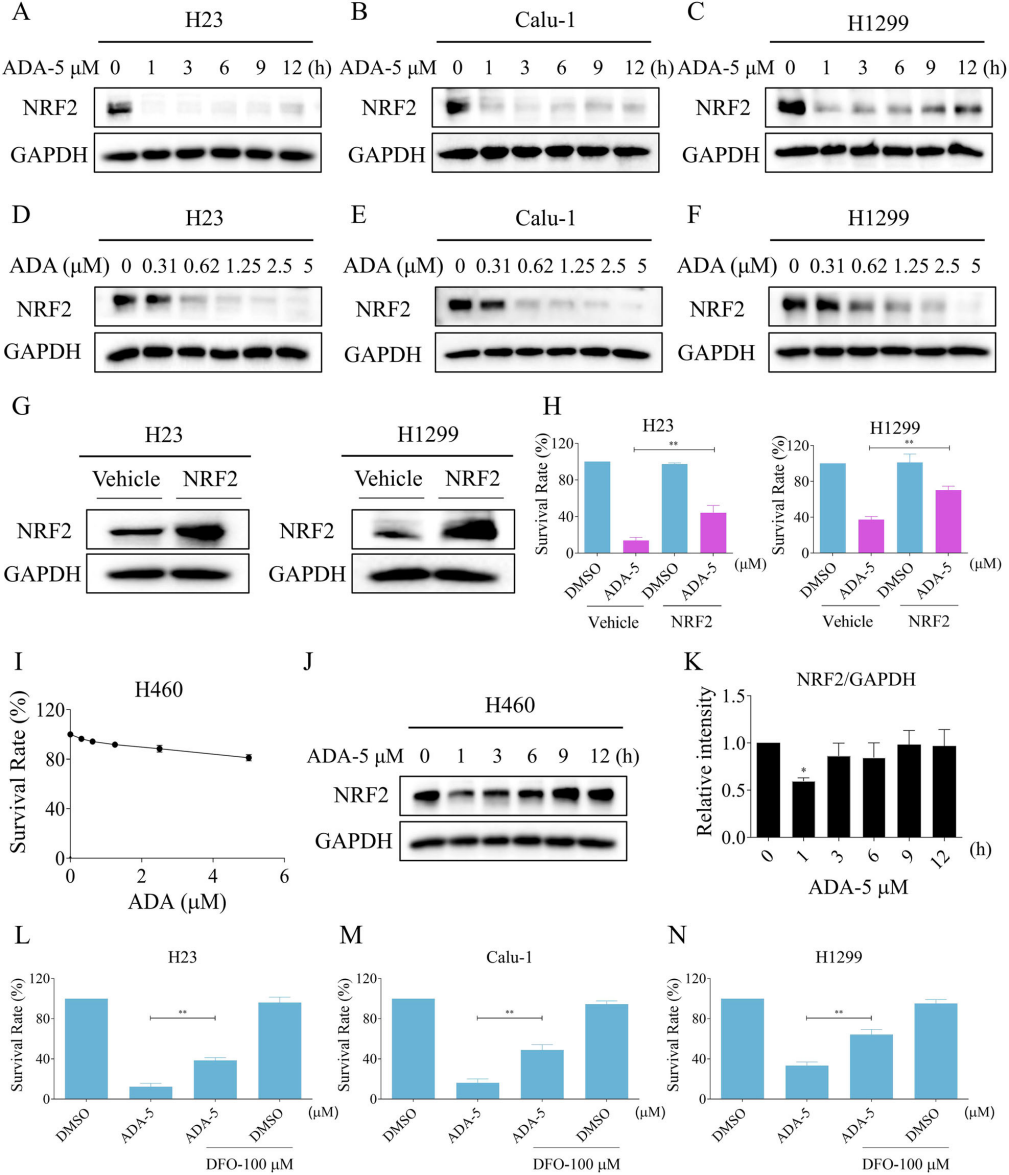
NRF2 inhibition plays a crucial role in ADA-induced cell death. **A**–**C** H23, Calu-1, and H1299 cells were exposed to ADA (5 μM) for specified durations, followed by Western blot to evaluate the expression of NRF2 and GAPDH. **D**–**F** H23, Calu-1, and H1299 cells were exposed to varying concentrations of ADA for 1 h, followed by Western blot to evaluate the expression of NRF2 and GAPDH. **G** H23 and H1299 cells were transfected with recombinant lentivirus, and the levels of NRF2 and GAPDH were subsequently measured. **H** H23 and H1299 cells were transfected with recombinant lentivirus. The cell survival rate was assessed after treatment with ADA (5 μM) for 24 h. **I** H460 cells were exposed to varying concentrations of ADA for 24 h, after which cell viability was assessed. **J**, **K** H460 cells were treated with ADA (5 μM) for specified durations, followed by Western blot to evaluate the expression of NRF2 and GAPDH. **L**–**N** H23, Calu-1, and H1299 cells were pre-incubated with DFO (100 μM) for 1 h, the cell survival rate was evaluated after treated with ADA (5 μM) for 24 h. *p < 0.05, **p < 0.01, as determined by One-way ANOVA.

A recent study found that sotorasib can covalently modify the functional cysteine of KEAP1 and induce the nuclear accumulation of NRF2 [13]. Tentatively, we examined the level of NRF2 in ADA-treated cells due to they have a similar mechanism of action between ADA and sotorasib. Surprisingly, ADA treatment significantly inhibited the expression of NRF2 in all cell lines, and exhibited an obvious correlation with concentration (Figs. 2A–F and S1A, B). To confirm the involvement of NRF2 in ADA-mediated cell death, we overexpressed the protein in H23 and H1299 cells. We observed that cells overexpressing NRF2 exhibited increased resistance to ADA compared to cells transfected with the vehicle (Fig. 2G, H). Moreover, we found that the KEAP1-mutated cell line H460 was resistant to ADA (Fig. 2I). Western blotting revealed that ADA treatment inhibited the expression of NRF2 in H460 cells at 1 h, but the reduction of NRF2 was transient (Fig. 2J, K). Previous studies have shown that the inhibition of NRF2 is closely related to ferroptosis [21, 22]. Therefore, we investigated the effects of a ferroptosis inhibitor on the anti-lung cancer activity of ADA. Our results indicate that the ferroptosis inhibitor deferoxamine mesylate (DFO) can significantly reverse the anti-lung cancer effects of ADA (Fig. 2L–N). This preliminary finding suggests that ADA may induce ferroptosis in lung cancer cells, though further investigation is needed to confirm the detailed molecular mechanisms involved. These results highlight the importance of NRF2 inhibition in ADA-induced cell death.

### ADA treatment promoted the ubiquitination of NRF2 in NSCLC cells

**Fig. 3:**
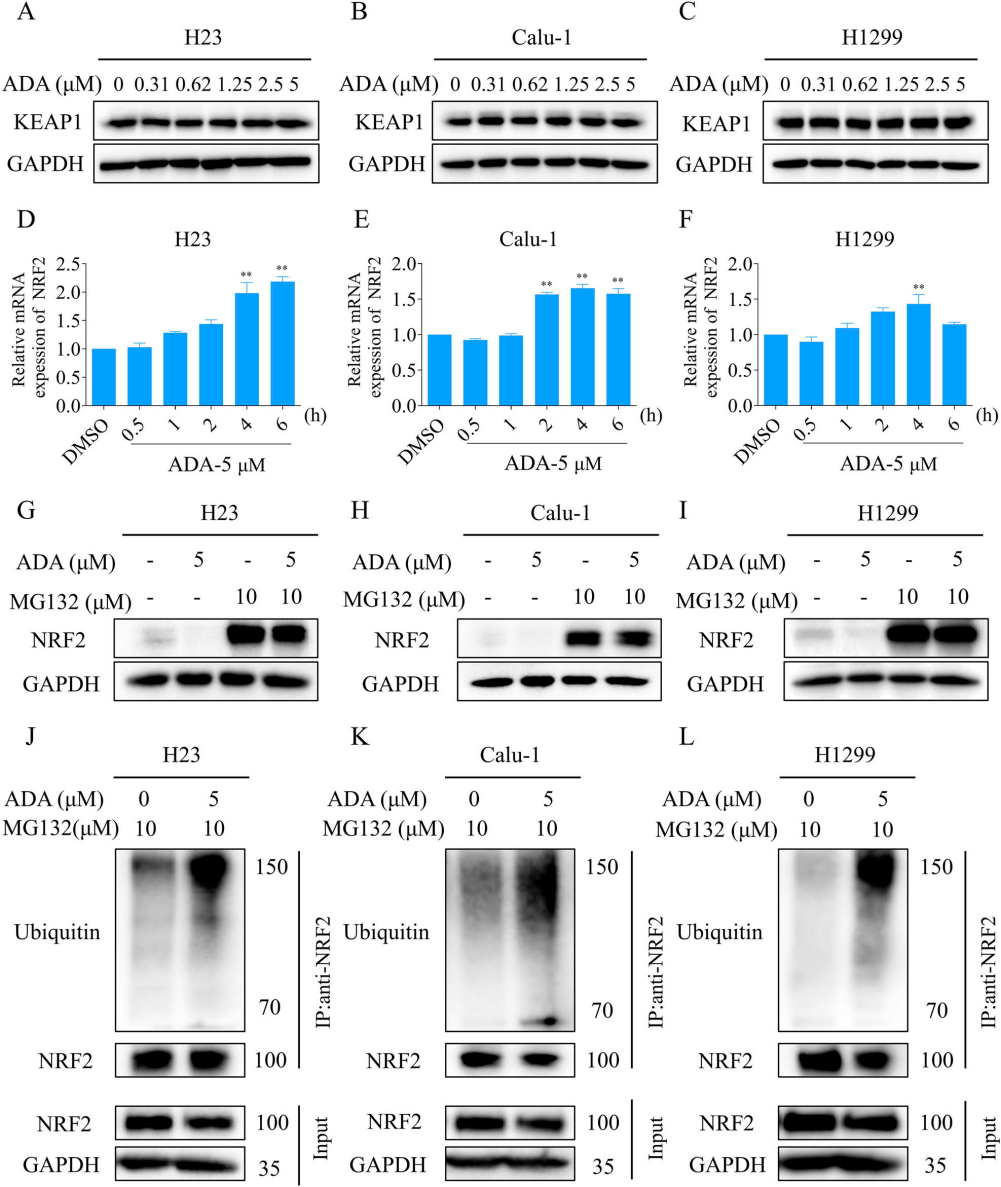
ADA treatment promoted the ubiquitination of NRF2 in NSCLC cells. **A–C** H23, Calu-1, and H1299 cells were exposed to ADA for 1 h, followed by Western blot to assess the expression of KEAP1 and GAPDH. **D**–**F** H23, Calu-1, and H1299 cells were exposed to ADA (5 μM) for specified durations, followed by qRT-PCR analysis to assess the mRNA levels of NRF2. **G**–**I** H23, Calu-1, and H1299 cells were pre-incubated with MG132 (10 μM) for 5 h. Subsequently, the expression of NRF2 and GAPDH was assessed by Western blot after treatment with ADA (5 μM) for 1 h. **J**–**L** H23, Calu-1, and H1299 cells were pre-incubated with MG132 (10 μM) for 5 h, followed by treatment with ADA (5 μM) for 1 h. The ubiquitination of NRF2 was assessed using an immunoprecipitation assay. **p < 0.01, as determined by One-way ANOVA.

We further explored the molecular mechanism by which ADA downregulates the level of NRF2 in NSCLC cells. Kelch-like ECH-associated protein 1 (KEAP1) is an important protein that plays a pivotal role in regulating the level of NRF2, ensuring that the balance of this key transcription factor is maintained for proper cellular function and response to oxidative stress [23]. Western blotting revealed that ADA treatment for 1 h did not result in a significant change in the level of KEAP1 in H23, Calu-1 and H1299 cells (Fig. 3A–C). Therefore, we next examined the mRNA level of NRF2 after ADA treatment. The qRT-PCR analysis revealed that the mRNA expression of NRF2 was not diminished but rather elevated following ADA treatment (Fig. 3D–F). This suggests that ADA does not exert its inhibitory effect on NRF2 by downregulating its mRNA level. The increase in NRF2 mRNA level after ADA treatment may reflect a compensatory response by the cells. Furthermore, we observed that the proteasome inhibitor MG132, at a concentration of 10 μM as per previous studies [24, 25], markedly reversed the ADA-induced inhibition of NRF2 protein, indicating that ADA may downregulate NRF2 expression by regulating its stability (Fig. 3G–I). Consistent with this notion, the ubiquitination of NRF2 was significantly increased in H23, Calu-1 and H1299 cells after ADA treatment (Fig. 3J–L).

### ADA can cooperate with cisplatin to induce cell death by inducing ROS accumulation in NSCLC cells

**Fig. 4:**
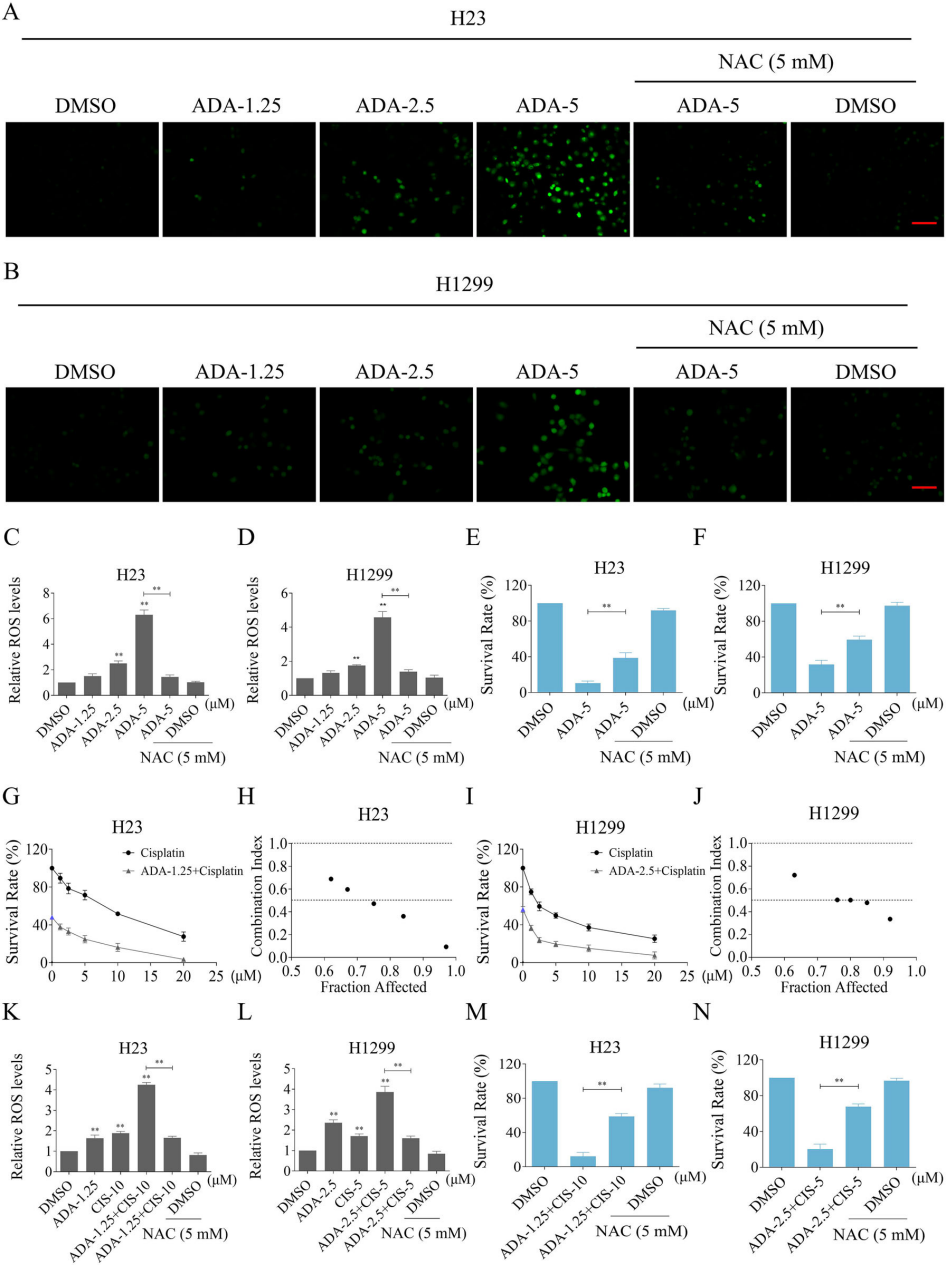
ADA can cooperate with cisplatin to induce cell death by inducing ROS accumulation in NSCLC cells. **A**, **B** H23 and H1299 cells were pre-incubated with NAC for 1 h, the ROS levels were assessed after treatment with ADA for 1 h. Scale bar = 100 μm. **C**, **D** Statistical evaluation of the fluorescence intensity shown in (**A**, **B**). **E**, **F** H23 and H1299 cells were pre-incubated with NAC for 1 h, the cell survival rate was evaluated after treatment with ADA (5 μM) for 24 h. **G**–**J** H23 and H1299 cells were treated with ADA and cisplatin (CIS) for 24 h, the cell survival rate was detected and the combination index (CI) between ADA and CIS was calculated based on the Chou-Talalay method. **K**, **L** H23 and H1299 cells were pre-incubated with NAC for 1 h, the ROS levels were assessed after treated with ADA and CIS for 1 h. **M**, **N** H23 and H1299 cells were pre-incubated with NAC for 1 h, the cell survival rate was evaluated after treated with ADA and CIS for 24 h. **p < 0.01, as determined by One-way ANOVA.

Inhibition of NRF2 may disrupt the cellular redox state and lead to an increase in the level of ROS [26, 27]. Indeed, we observed that silencing NRF2 significantly elevated ROS level in H23 and H1299 cells (Fig. S2A–D). Moreover, ADA treatment also resulted in a significant increase in ROS level in both H23 and H1299 cells, with the effect showing a clear concentration-dependent correlation (Fig. 4A–D). To identify the impact of ROS in ADA-induced cytotoxicity, the ROS scavenger N-acetylcysteine (NAC) was employed at a concentration of 5 mM, as used in previous studies [28, 29]. We noted that NAC pretreatment substantially reduced the ADA-caused accumulation of cellular ROS in H23 and H1299 cell lines (Fig. 4A–D). Notably, the cell growth inhibition caused by ADA was also reversed by NAC (Fig. 4E, F). These findings suggest that ADA inhibits NRF2 and upregulates ROS level, thereby exerting its anti-tumor effects.

Recent studies have indicated that NRF2 is abnormally upregulated in various tumor cells, thereby promoting tumor cell growth and drug resistance [30, 31]. Therefore, it’s interesting to explore new drug combinations based on ADA. Indeed, we found that ADA can cooperate with cisplatin to promote cell death in H23 and H1299 cell lines (Fig. 4G–J). Combination index analysis revealed that ADA in combination with cisplatin demonstrated a pronounced synergistic effect in inhibiting cell growth (Fig. 4G–J). Previous studies have revealed that ROS accumulation is pivotal in mediating the antitumor efficacy of platinum drugs [32–34]. Consistently, we found that cisplatin treatment upregulated the ROS level in H23 and H1299 cell lines, and the increase in ROS level was more significant when cisplatin was used in combination with ADA (Fig. 4K, L). Notably, the increase in ROS level and subsequent cell death were substantially attenuated by NAC pretreatment, indicating that ROS upregulation is involved in the cooperative antitumor effects of ADA and cisplatin (Fig. 4K–N).

### The phosphorylation of AKT was increased in NSCLC cells after ADA treatment

**Fig. 5:**
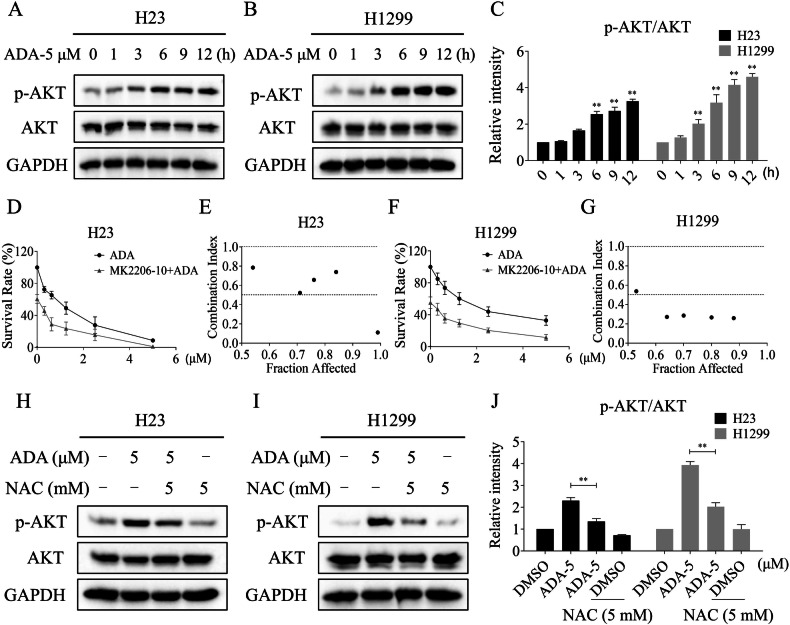
The phosphorylation of AKT was increased in NSCLC cells after ADA treatment. **A**–**C** H23 and H1299 cells were treated with ADA (5 μM) for specified durations, followed by Western blot to evaluate the expression of p-AKT, AKT and GAPDH. **D**–**G** H23 and H1299 cells were cultured with ADA and MK2206 for 24 h, the cell survival rate was detected and the combination index (CI) between ADA and MK2206 was calculated based on the Chou-Talalay method. **H**–**J** H23 and H1299 cells were pre-incubated with NAC for 1 h. Subsequently, the expression of p-AKT, AKT, and GAPDH was assessed after treatment with ADA (5 μM) for 9 h. **p < 0.01, as determined by One-way ANOVA.

Although ADA has shown excellent antitumor activity in KRASG12C-mutated tumors, the clinical utility of ADA is constrained by the emergence of drug resistance. Recent studies have revealed that PI3K/AKT pathway activation confers resistance to clinical KRASG12C inhibitors [11, 35, 36]. Our previous research found that an increase in ROS can lead to compensatory activation of the AKT pathway in tumor cells [37]. Therefore, we examined the phosphorylation of AKT in H23 and H1299 cells. Western blot showed that the expression of p-AKT was markedly increased following ADA treatment (Fig. 5A–C). To identify the impact of p-AKT in ADA-induced cytotoxicity, the AKT-specific inhibitor MK2206 was used. We observed that MK2206 significantly increased the antitumor activity of ADA in H23 and H1299 cells (Fig. 5D–G). Notably, the increased expression of p-AKT evoked by ADA was significantly blocked by NAC pretreatment (Fig. 5H–J). These findings suggest that the AKT pathway upregulation was a compensatory response to the accumulation of ROS elicited by ADA.

### ADA treatment activated autophagy in NSCLC cells

**Fig. 6:**
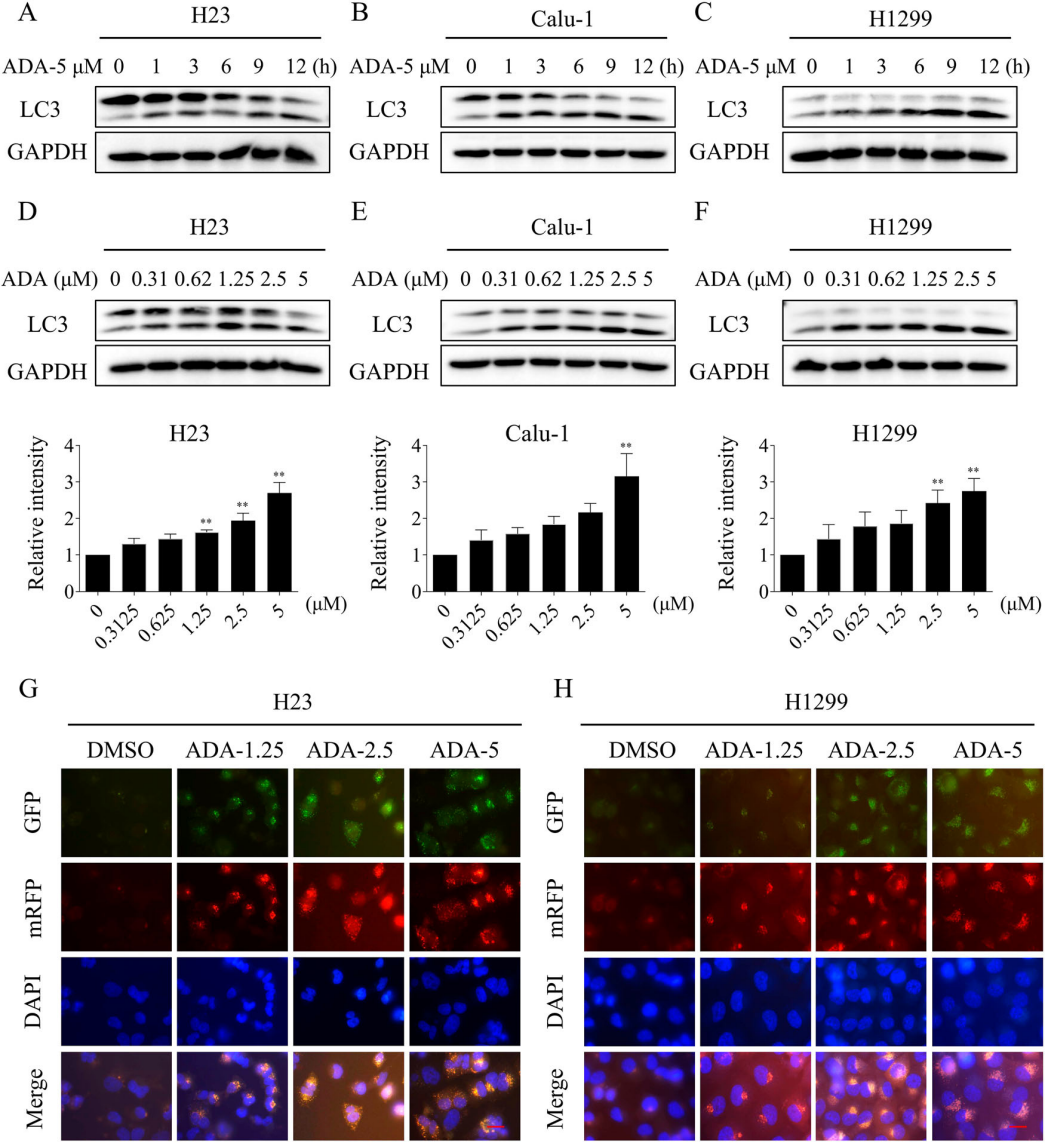
ADA treatment activated autophagy in NSCLC cells. **A**–**C** H23, Calu-1, and H1299 cells were treated with ADA (5 μM) for specified durations, followed by Western blot to assess the expression of LC3 and GAPDH. **D**–**F** H23, Calu-1, and H1299 cells were exposed to varying concentrations of ADA for 1 h, followed by Western blot to assess the expression of LC3 and GAPDH. **G**, **H** H23 and H1299 cells were transfected with lentivirus, images were captured using a fluorescence microscope after treatment with varying concentrations of ADA for 9 h. The autophagic flux can be assessed by comparing the number of GFP (green fluorescence)/mRFP (red fluorescence) double-positive vesicles. Scale bar = 25 μm.**p < 0.01, as determined by One-way ANOVA.

**Fig. 7:**
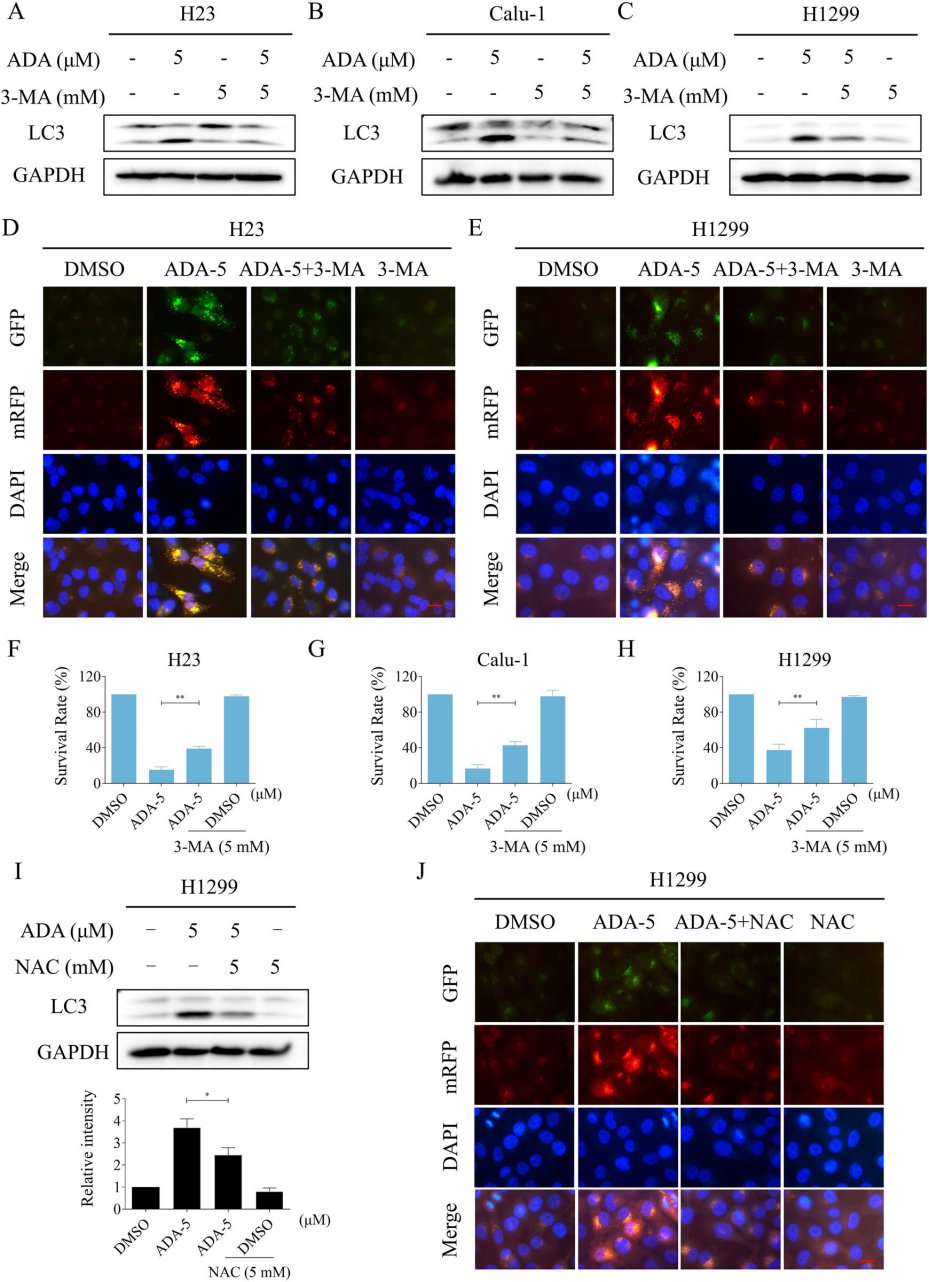
The induction of autophagy by ADA is crucial for its antitumor activity. **A**–**C** H23, Calu-1, and H1299 cells were pre-incubated with 3-MA for 1 h, the expression levels of LC3 and GAPDH were detected after treatment with ADA (5 μM) for 1 h. **D**, **E** H23 and H1299 cells were transfected with lentivirus and then pre-incubated with 3-MA for 1 h, images were captured using a fluorescence microscope after treatment with ADA (5 μM) for 9 h. **F**–**H** H23, Calu-1 and H1299 cells were pre-incubated with 3-MA for 1 h, the cell survival rate was detected after treatment with ADA (5 μM) for 24 h. **I** H1299 cells were pre-incubated with NAC for 1 h, the expression levels of LC3 and GAPDH were detected after treatment with ADA (5 μM) for 1 h. **J** H1299 cells were transfected with lentivirus and then pre-incubated with NAC for 1 h, images were captured using a fluorescence microscope after treatment with ADA (5 μM) for 9 h. Scale bar = 25 μm. *p < 0.05, **p < 0.01, as determined by One-way ANOVA.

Previous studies have revealed that autophagy is a downstream effector of ROS, and can be activated by ROS inducers [38–40]. The LC3-II/I ratio is often used as a marker to monitor autophagic activity within cells [41]. Consistently, we found that the ratio of LC3-II/I was substantially enhanced following ADA treatment in H23, Calu-1 and H1299 cells, and exhibited a positive correlation with ADA concentration (Fig. 6A–F). Immunofluorescence experiment further identified that autophagy was activated after ADA treatment (Fig. 6G, H). Notably, the autophagy inhibitor 3-methyladenine (3-MA) effectively counteracts both the activation of autophagy and the growth inhibitory effect induced by ADA in H23, Calu-1 and H1299 cells (Fig. 7A–H).

We next set out to determine the relationship between autophagy and ROS generation. Western blot revealed that NAC pretreatment markedly suppressed the ADA-induced autophagy in H1299 cells (Fig. 7I). Furthermore, immunofluorescence staining showed that the ADA-induced activation of autophagy was also reversed by NAC pretreatment (Fig. 7J).

### ADA in combination with panobinostat elicits significant synthetic lethality in NSCLC cells

**Fig. 8:**
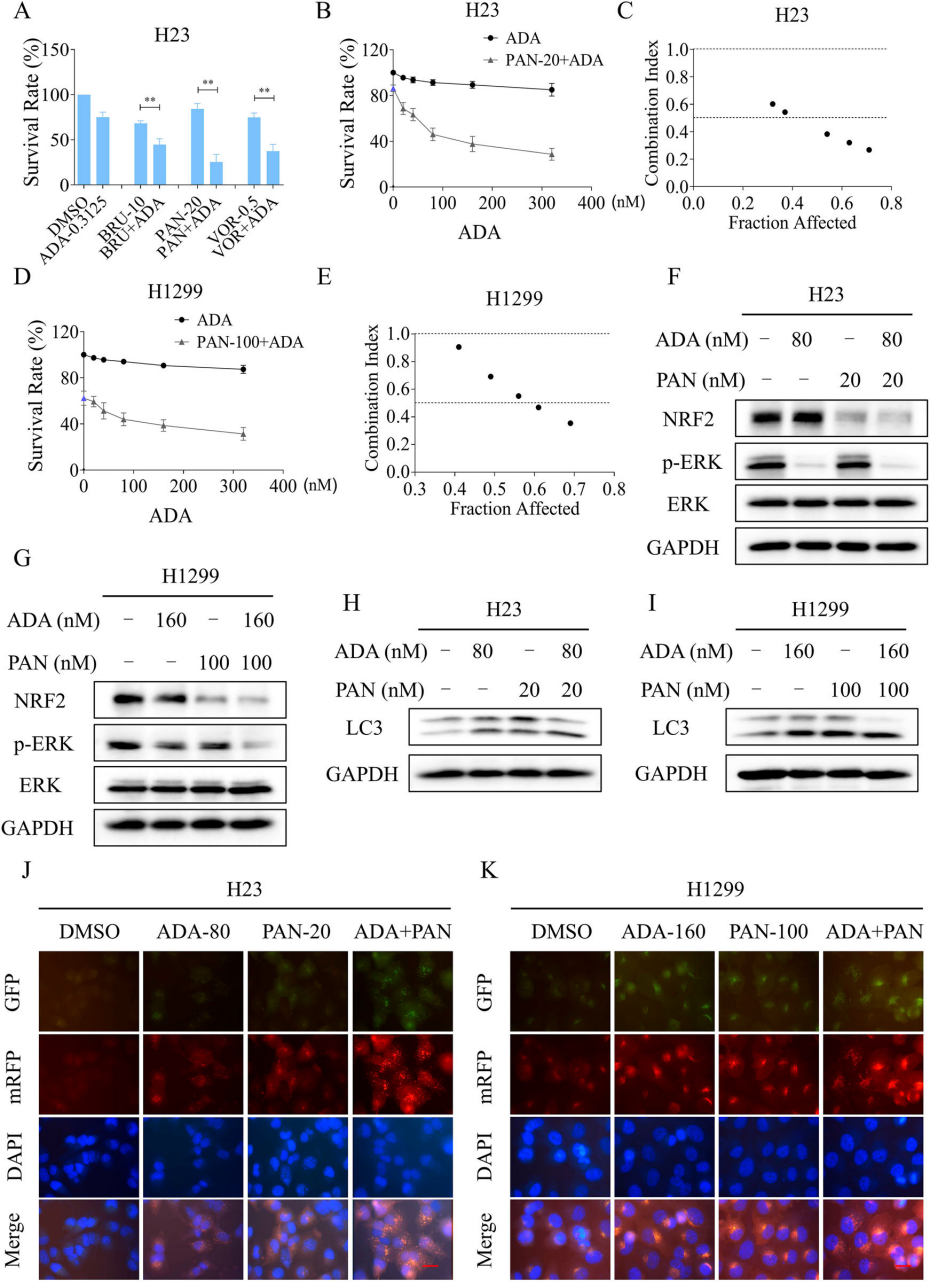
ADA in combination with PAN elicits significant synthetic lethality in NSCLC cells. **A** H23 cells were treated with ADA (0.3125 μM) alone or in combination with BRU (10 nM), PAN (20 nM) and VOR (0.5 μM) for 24 h, after which cell viability was assessed. **B**–**E** H23 and H1299 cells were treated with PAN alone or in combination with various concentrations of ADA (20, 40, 80, 160, 320 nM) for 24 h, after which cell viability and related CI values were assessed. **F**–**H** H23 and H1299 cells were treated with ADA or PAN alone, or in combination for 3 h, the associated proteins were detected by Western blot. **I** H1299 cells were treated with ADA or PAN alone, or in combination for 9 h, the associated proteins were detected by Western blot. **J**, **K** H23 and H1299 cells were transfected with lentivirus, images were captured using a fluorescence microscope after treated with ADA or PAN alone, or in combination for 9 h. Scale bar = 25 μm. **p < 0.01, as determined by Two-way ANOVA.

The above studies indicate that the inhibition of NRF2 is pivotal in mediating the antitumor efficacy of ADA. However, it can also lead to the activation of cellular compensation pathways, such as the AKT pathway, particularly at high concentrations. Considering that ADA can significantly inhibit the ERK pathway at very low concentrations, it is interesting to explore its combination with NRF2 pathway inhibitors. Brusatol, a compound isolated from the *Brucea javanica* plant, is known as an NRF2 inhibitor [42]. In addition, HDAC inhibitors such as MS-275, vorinostat, belinostat, and panobinostat can significantly inhibit NRF2 to exert their anti-tumor activity [43–45]. Indeed, the expression of NRF2 in H23 cells was significantly reduced following treatment with brusatol (BRU), vorinostat (VOR), or panobinostat (PAN) (Fig. S3A, B). Cell viability assays indicated that these agents markedly increased the sensitivity of H23 cells to ADA (Fig. 8A). To accelerate clinical implementation, we opted to explore the combined efficacy and mechanism of PAN with ADA. The combination index results indicate that the combination of ADA and PAN elicits significant synthetic lethality in H23 and H1299 cells (Fig. 8B–E). Western blotting showed that the levels of p-ERK and NRF2 were significantly inhibited after the combination treatment in H23 and H1299 cells (Fig. 8F, G).

**Fig. 9:**
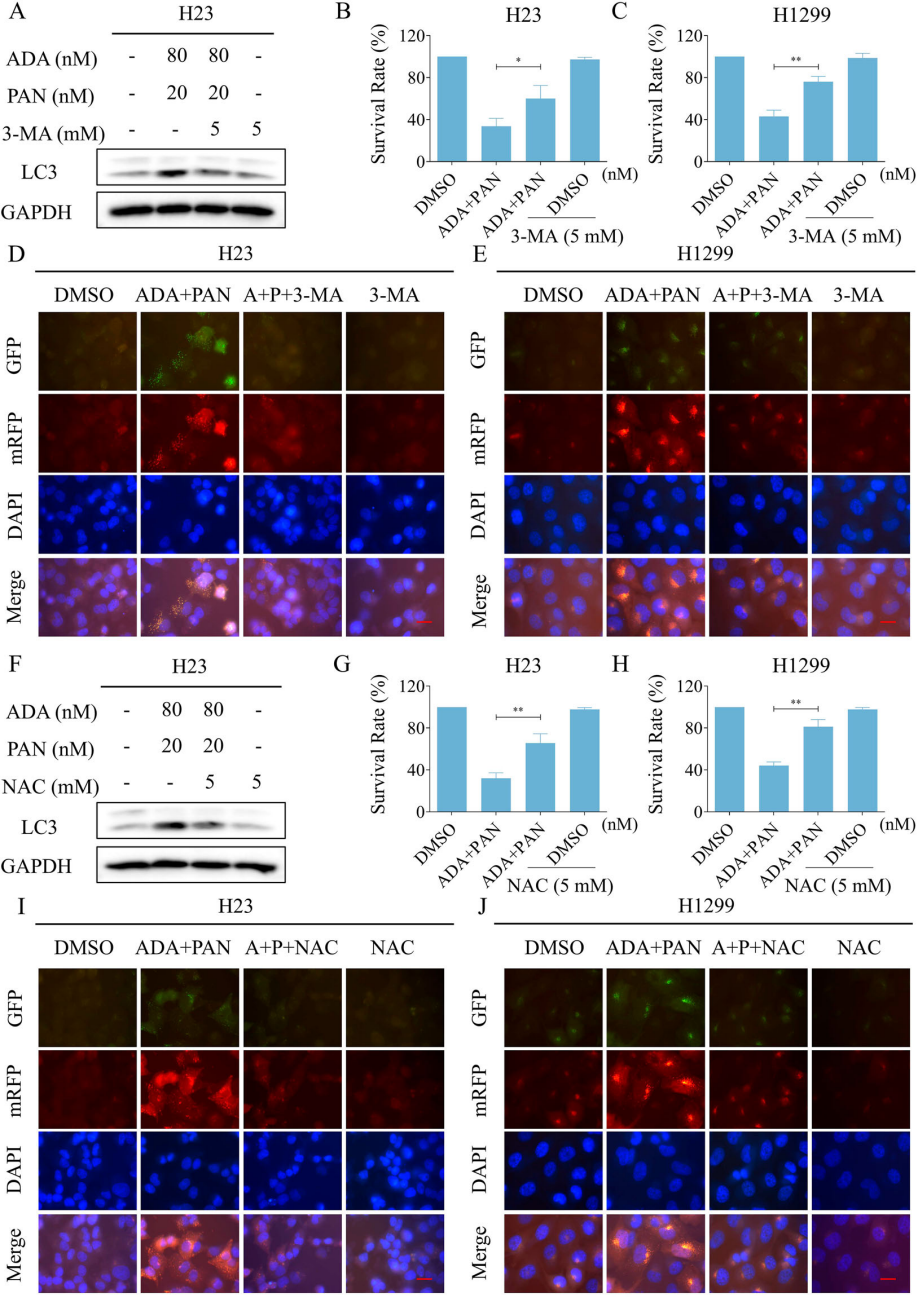
The induction of autophagy by the combination is critical for its antitumor activity. **A** H23 cells were pre-incubated with 3-MA for 1 h, the expression of LC3 and GAPDH was detected by Western blot after treated with a combination of ADA (80 nM) and PAN (20 nM) for 9 h. **B**, **C** H23 and H1299 cells were pre-incubated with 3-MA for 1 h, the cell survival rate was evaluated after treatment with ADA (80 nM for H23 cells, 160 nM for H1299 cells) and PAN (20 nM for H23 cells, 100 nM for H1299 cells) for 24 h. **D**, **E** H23 and H1299 cells were transfected with lentivirus and then pre-incubated with 3-MA for 1 h, images were captured using a fluorescence microscope after treatment with ADA (80 nM for H23 cells, 160 nM for H1299 cells) and PAN (20 nM for H23 cells, 100 nM for H1299 cells) for 9 h. **F** H23 cells were pre-incubated with NAC for 1 h, the expression levels of LC3 and GAPDH were detected by Western blot after treatment with ADA (80 nM) and PAN (20 nM) for 9 h. **G**, **H** H23 and H1299 cells were pre-incubated with NAC for 1 h, the cell survival rate was assessed after treatment with ADA (80 nM for H23 cells, 160 nM for H1299 cells) and PAN (20 nM for H23 cells, 100 nM for H1299 cells) for 24 h. **I**, **J** H23 and H1299 cells were transfected with lentivirus and then pre-incubated with NAC for 1 h, images were captured using a fluorescence microscope after treatment with ADA (80 nM for H23 cells, 160 nM for H1299 cells) and PAN (20 nM for H23 cells, 100 nM for H1299 cells) for 9 h. Scale bar = 25 μm. *p < 0.05, **p < 0.01, as determined by One-way ANOVA.

We conducted further investigation into the downstream molecular mechanism contributing to the combined effect of ADA and PAN. Western blot revealed that the ratio of LC3-II/I increased after ADA or PAN treatment, while a more significant increase observed in the combination treatment group (Fig. 8H, I). Immunofluorescence experiment further identified that ADA can cooperate with PAN to activate autophagy in H23 and H1299 cells (Fig. 8J, K). Notably, 3-MA can effectively block both the activation of autophagy and the growth inhibitory effect induced by the combination in H23 and H1299 cells, suggesting that autophagy is pivotal in the synergistic antitumor activity of ADA and PAN (Fig. 9A–E). Moreover, the activation of autophagy and the growth inhibitory effect elicited by ADA and PAN was also attenuated by NAC pretreatment (Fig. 9F–J).

### ADA can cooperate with PAN to inhibit tumor growth in vivo

**Fig. 10:**
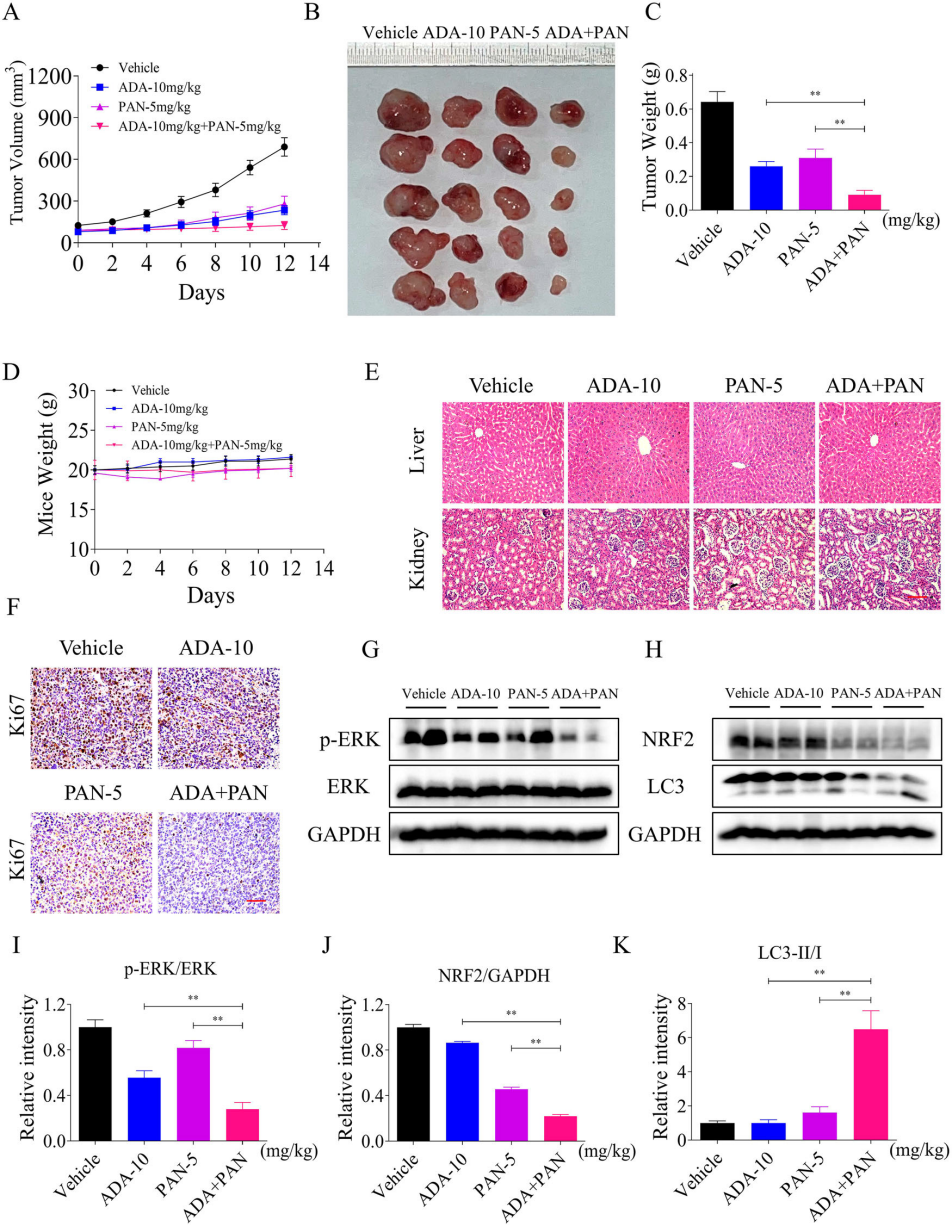
ADA can cooperate with PAN to inhibit tumor growth in vivo. **A** H1299 cells were subcutaneously injected into nude mice, and tumor volumes were measured in the vehicle, ADA, PAN, and combined ADA and PAN treatment groups. **B** An image of tumors for different groups was captured. **C** The weights of tumors for different groups were measured. **D** The body weights of nude mice in different groups were measured. **E** HE staining was performed on liver and kidney tissues in different groups. **F** Ki67 levels in the tumors were measured in different groups. **G**–**K** The expression of NRF2, LC3, p-ERK, ERK and GAPDH was evaluated by Western blot. **p < 0.01, as determined by One-way ANOVA.

To accelerate clinical application, we further explored the anti-tumor activity of ADA in combination with PAN in vivo. Nude mouse experiments demonstrated that both ADA and PAN can significantly inhibit tumor growth in vivo, but the combination therapy exhibits superior inhibitory activity (Fig. 10A–C). The body weight of nude mice and HE staining experiments indicated that the combination therapy group is well-tolerated (Fig. 10D, E). Immunohistochemistry assays revealed a significant inhibition of Ki67 levels in the tumors by the combination therapy (Fig. 10F). Notably, ADA in combination with PAN can significantly inhibit the expression of p-ERK and NRF2 while upregulating the LC3-II/I ratio in vivo, consistent with the in vitro experiments (Fig. 10G–K).

## Discussion

Adagrasib, a potent KRASG12C inhibitor, has demonstrated significant antitumor efficacy in patients with KRASG12C-mutated NSCLC [46, 47]. Previous studies have mainly focused on the resistance mechanisms of adagrasib, and revealing that both genetic and nongenetic mechanisms can promote cancer cell resistance to adagrasib [5, 35]. Noritaka Tanaka et al. found that a patient who developed resistance to adagrasib exhibited 10 alterations in four genes [7]. Moreover, a research by Takamasa Koga et al. revealed multiple secondary mutations in the KRAS gene that promote adagrasib resistance [9]. However, the off-target effects and their underlying mechanisms of adagrasib remain unclear. Here, we found that adagrasib treatment markedly inhibited the growth of cells harboring the KRASG12C mutation. Interestingly, the non-KRASG12C cell lines H1299 and PC-9 also exhibited sensitivity to adagrasib, suggesting that the drug exerts off-target effects. Mechanistically, adagrasib treatment reduced NRF2 protein level by promoting its ubiquitination, thereby increasing intracellular ROS level and subsequently activating autophagy. Notably, adagrasib significantly inhibited ERK phosphorylation at low concentrations. However, ERK phosphorylation gradually increased with escalating concentrations of adagrasib. This finding suggests that higher adagrasib levels may induce off-target effects, possibly through NRF2 inhibition and the consequent accumulation of ROS, which could activate compensatory ERK signaling. Nevertheless, further investigation is needed to validate this hypothesis.

Regarding NRF2, we found that after adagrasib treatment, the protein expression level of NRF2 in NSCLC cells significantly decreased, while its mRNA level increased. Further investigation revealed that adagrasib treatment led to an increase in the ubiquitination of NRF2. These findings suggest that adagrasib induces the ubiquitination and degradation of NRF2, thereby reducing its protein levels. Additionally, we observed that NRF2 expression was significantly inhibited in NSCLC cells 1 h after adagrasib treatment. However, with prolonged treatment, NRF2 expression gradually increased. These results indicate that the rise in NRF2 mRNA level following adagrasib treatment may represent a compensatory response by the cells. Further studies are needed to confirm the underlying molecular mechanisms.

To elucidate the role of NRF2 inhibition in the antitumor effects of adagrasib, we overexpressed NRF2 in NSCLC cells. Our findings showed that NRF2 overexpression reversed the adagrasib-induced cell death in H23 and H1299 cells. Notably, the KEAP1-mutated cell line H460 showed significantly resistant to adagrasib. This finding is consistent with recent research that KEAP1 mutation is an independent determinant of poor clinical outcomes in patients with KRASG12Ci monotherapy [48]. To explore the underlying mechanism, we further examined the expression of NRF2 in H460 cells after adagrasib treatment. Western blot revealed that adagrasib treatment resulted in a downregulation of NRF2 in H460 cells, but the suppressive impact of adagrasib on NRF2 was transient. These findings suggest that the suppressive activity of adagrasib on NRF2 is independent of the KRASG12C mutation, and that NRF2 inhibition is crucial for the cytotoxicity of adagrasib. Interestingly, we found that the ferroptosis inhibitor DFO can reverse the anti-tumor activity of adagrasib, which is consistent with previous studies indicating that inhibition of NRF2 can induce cell ferroptosis [49, 50]. However, further investigation is needed to understand how adagrasib induces ferroptosis and to explore the detailed molecular mechanisms involved. Additionally, the molecular targets through which adagrasib regulates NRF2 expression remain to be further explored.

ROS is an important metabolic product in cells. Previous studies have revealed that some clinical drugs such as cisplatin, regorafenib and lenvatinib can upregulate intracellular ROS level to trigger tumor cell death [51–53]. In addition, radiotherapy and photodynamic therapy can also induce ROS-dependent cell death in tumor cells [54–56]. NRF2 is vital for regulating cellular redox balance, inhibition of NRF2 may disrupt the cellular redox state and lead to an increase in the level of ROS. Indeed, we found that silencing NRF2 or treating with adagrasib led to a significant elevation in ROS levels. Moreover, the cell growth inhibition caused by adagrasib was reversed by NAC, indicating that ROS upregulation is pivotal for the antitumor activity of adagrasib. Importantly, we found adagrasib can cooperate with cisplatin to induce cell death in H23 and H1299 cells. Further investigation suggests that ROS upregulation is vital for the synergistic antitumor efficacy of adagrasib and cisplatin. Our findings further corroborate the idea that modulation of ROS level in tumor cells represents a promising strategy for antitumor intervention. Despite these findings, more investigations are required to identify the detailed molecular mechanism and the in vivo antitumor efficacy of adagrasib and cisplatin.

Another key finding of our study is that autophagy is activated in response to adagrasib, and this activation is crucial for the antitumor activity of adagrasib. The LC3-II/I ratio is commonly used as a marker to monitor autophagic activity within cells. Consistently, we found that the LC3-II/I ratio was markedly increased following adagrasib treatment in NSCLC cells. Notably, the activation of autophagy evoked by adagrasib was significantly blocked by NAC pretreatment, indicating that autophagy serves as a downstream effector of ROS. Moreover, we found that the expression of p-AKT was also markedly increased following adagrasib treatment in H23 and H1299 cells. However, further analysis revealed that the AKT pathway upregulation was a compensatory response to the accumulation of ROS elicited by adagrasib. This result provides new insights for a deeper knowledge of the mechanisms of adagrasib resistance.

To accelerate clinical translation and application, we further explored the anti-tumor activity of adagrasib in combination with NRF2 pathway inhibitors. The Western blot results showed that brusatol, vorinostat, and panobinostat significantly inhibited NRF2 expression in NSCLC cells. Further research revealed that all three compounds enhanced the sensitivity of H23 cells to adagrasib. Among them, panobinostat has been approved for the clinical treatment of multiple myeloma and is currently undergoing clinical trials for other solid tumors, including lung cancer and colorectal cancer, demonstrating promising anti-tumor potential [14, 15]. Therefore, considering its broad anti-tumor activity and clinical applicability, we chose to combine panobinostat with adagrasib for further investigation. Notably, adagrasib can collaborate with panobinostat to suppress tumor growth in vivo. Western blot revealed that adagrasib in combination with panobinostat can markedly inhibit the expression of p-ERK and NRF2, while increasing the LC3-II/I ratio both in vitro and in vivo. Nevertheless, the detailed molecular mechanism of how panobinostat regulates NRF2 expression still requires further exploration, and its clinical activity in combination with adagrasib needs further confirmation.

## Conclusions

In summary, we identified a novel mechanism of adagrasib, which will bring potential benefits for the clinical use of adagrasib. While more work is needed to analyze the patients treated with adagrasib and to confirm the potential targets and mechanisms of this drug. Moreover, our findings suggest that the combination of adagrasib and panobinostat exhibits strong synergistic anti-lung cancer activity. These results furnish a robust theoretical basis for the further exploration and application of this combination regimen in clinical settings.

## Materials and methods

### Reagents

Adagrasib, MK-2206, 3-methyladenine and cisplatin were procured from TargetMol (Shanghai, China). Panobinostat, deferoxamine mesylate and vorinostat were obtained from Aladdin Industrial Corporation (Shanghai, China). Anti-NRF2 (16396-1-AP), anti-LC3 (14600-1-AP), anti-KEAP1 (10503-2-AP) and anti-GAPDH (10494-1-AP) antibodies were procured from Proteintech Group (Wuhan, China). Antibodies against p-ERK (4370), ERK (4695), p-AKT (4060) and AKT (2920) were procured from Cell Signaling Technology (Danvers, USA).

### Cell culture and viability assay

H23, H1299, PC-9 and Calu-1 cells were acquired from Chinese Academy of Sciences (Shanghai, China). H23, H1299 and PC-9 cells were maintained in RPMI 1640 or DMEM medium, while Calu-1 cells were cultured in McCoy’s 5A medium at 37 °C. All media were enriched with 10% fetal bovine serum and then placed in a 5% CO_2_ incubator. For cell viability assay, the cells were inoculated onto a six-well plate and incubated with a compound at 37°C for 24 h. Ultimately, cell viability was assessed, and the combination index (CI) was calculated using the Chou-Talalay method [57].

### Immunoprecipitation and Western blot analysis

After the indicated treatment, the cells were lysed to obtain a total cell lysate. The protein concentration was measured and an appropriate amount of the corresponding antibody was added to the lysate. The mixture was incubated overnight at 4°C and then mixed with agarose beads for 4 h. After washing with lysis buffer, they were prepared for Western blot analysis. The samples were carefully transferred to a PVDF membrane, and to minimize non-specific binding, the membrane was then blocked with a 5% skim milk solution for a period of 1 to 2 h, ensuring optimal conditions for subsequent analysis. After blocking, the membranes were rinsed with TBST three times for 5 min each. The membranes were incubated with primary antibodies overnight at 4°C, followed by a 1-h incubation with an appropriate secondary antibody. Finally, the membranes were imaged using ECL chemiluminescent reagents and the signal intensity was quantified using ImageJ software.

### Determination of ROS level

To measure intracellular ROS level, the cells were seeded in six-well plates. After the indicated treatment, the cells were incubated with the DCFH-DA probe for 30 min. Then the cells were rinsed thoroughly with PBS, and the intracellular ROS level was visualized using fluorescence microscopy.

### Immunofluorescence assay

After the indicated treatment, the cells were rinsed with PBS three times to ensure the removal of any residual substances. Following this rinsing procedure, the cells were fixed with 4% paraformaldehyde for 15 min. A permeabilization solution was carefully prepared and subsequently applied, allowing the cells to be permeabilized for a duration of half an hour at room temperature, ensuring optimal conditions for the experiment. Subsequently, they were rinsed with PBS and then blocked with a BSA solution for 1 h to minimize non-specific interactions. After this blocking period, they were incubated overnight with the primary antibody at 4 °C, allowing for optimal binding and specificity. Following incubation, the cells were washed several times with PBST, incubated with the secondary antibody in a wet box for 90 min, stained with DAPI, and finally observed under a microscope.

### Quantitative real-time PCR assay

Following the treatment of the cells with the compound, total RNA was meticulously extracted utilizing TRIzol reagent from Invitrogen (USA). Subsequently, the extracted RNA was converted into cDNA through the synthesis process, which employed the Kit provided by TaKaRa (Japan). The relative level of a specific gene was determined by qRT-PCR analysis, with GAPDH serving as the internal control. The sequences of the primers utilized are as follows: NRF2 (forward primer: TCCAGTCAGAAACCAGTGGAT, reverse primer: GAATGTCTGCGCCAAAAGCTG); GAPDH (forward primer: AGCCACATCGCTCAGACAC, reverse primer: GCCCAATACGACCAAATCC).

### Lentivirus transfection

The RFP-GFP-tagged LC3 lentivirus was obtained from GeneChem (Shanghai, China). The cells were maintained in a 12-well plate for 24 h, followed by the addition of an appropriate virus sensitization solution and lentivirus. After 36 h, the medium was changed to fresh medium. To assess autophagic status, the cells were cultured with designated agents for specified durations. Following treatment, images were captured using a fluorescence microscope.

### Xenograft experiments

The xenograft experiments were performed in full adherence to the guidelines for the Care and Use of Laboratory Animals. After subcutaneous injection of H1299 cells into female nude mice (6 weeks old), the mice were randomly assigned to four groups of five mice each once the tumor volume reached approximately 100 mm^3^, according to our prior experience. Blinding was not performed during the drug treatment process. The administration protocol is as follows: vehicle, adagrasib (10 mg/kg, administered intragastrically), panobinostat (5 mg/kg, administered intraperitoneally) and a combination of adagrasib (10 mg/kg, administered intragastrically) and panobinostat (5 mg/kg, administered intraperitoneally). This regimen is administered once every two days. In the end, administer euthanasia to the mice and meticulously extract the tumors and organs. HE staining was further utilized to identify and assess the pathological changes of organs.

### Statistical analyses

Data analysis was performed using GraphPad Prism 5.0 software. Results are expressed as mean ± SD. One-way ANOVA or Two-way ANOVA was employed to identify significant differences between groups, with a *p* value of less than 0.05 deemed statistically significant.

## Supplementary information

Supporting Information

Original Western Blots

## Data Availability

The data are available upon request by contacting the corresponding authors.
