# Supplementary material for: Panobinostat potentiates adagrasib-induced cell death by triggering autophagy in human non-small cell lung cancer
Source: Cell Death Discov. 2025 Aug 1;11:360. doi: 10.1038/s41420-025-02657-9 (PMC12316882; doi:10.1038/s41420-025-02657-9)
Supplement: Supplementary file 1 — Supporting Information [file 41420_2025_2657_MOESM1_ESM.docx]

**Supporting Information**

**Panobinostat Potentiates Adagrasib-Induced Cell Death by Triggering Autophagy in Human Non-Small Cell Lung Cancer**

Hui Lu^1,2#^, Wenying Fu^2#^, Yiqun Xia^3#^, Ying Yan^2^, Chongchong Shu^2^, Yinghua Chen^2^, Chenxin Xu^2^, Peisen Zheng^2^, Xin Shen^2^, Ri Cui^2^*, Peng Zou^1,2^*, Daoyong Ni^1^*

^1^ Affiliated Yueqing Hospital, Wenzhou Medical University, Wenzhou, China

^2^ School of Pharmaceutical Sciences, Wenzhou Medical University, Wenzhou, China

^3^ The First Affiliated Hospital of Wenzhou Medical University, Wenzhou Medical University, Wenzhou, China

* Corresponding author: Daoyong Ni: daoyongni@163.com

* Corresponding author: Peng Zou: zoupeng@wmu.edu.cn

* Corresponding author: Ri Cui: wzmucuiri@163.com

Co-authors' email addresses: Hui Lu: 2537701900@qq.com; Wenying Fu: 17816786727@163.com; Yiqun Xia: [yiqunxia@yeah.net](mailto:yiqunxia@yeah.net); Ying Yan: 2625316840@qq.com; Chongchong Shu: 2089858841@qq.com; Yinghua Chen: 1547765895@qq.com; Chenxin Xu: 3240225953@qq.com; Peisen Zheng: [980406643@qq.com](mailto:980406643@qq.com); Xin Shen: 1114239370@qq.com

# These authors contributed equally to the article

**Figure S1**

**
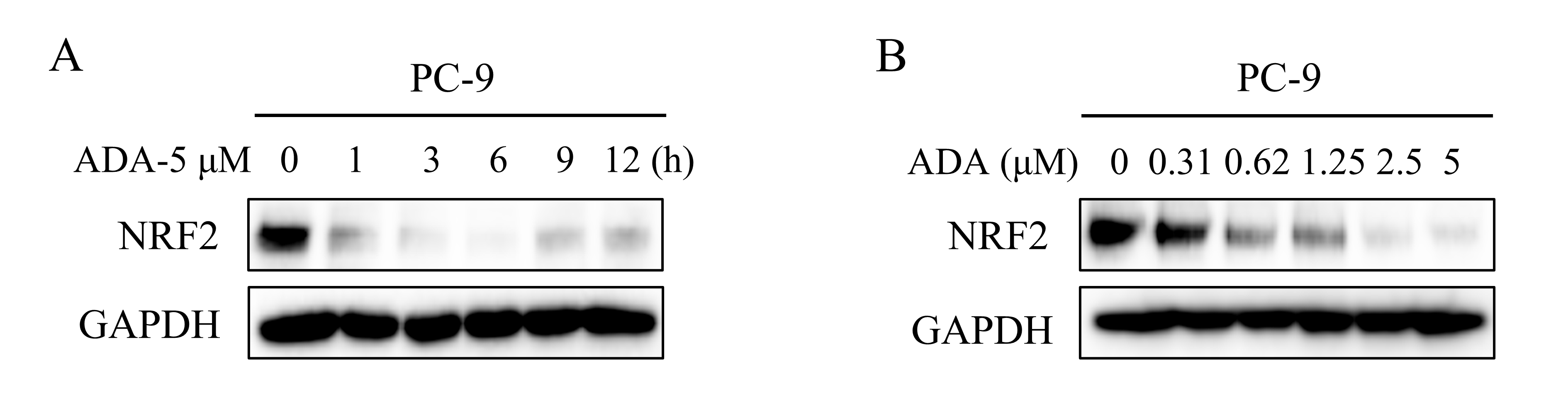
**

**Figure S1: Adagrasib (ADA) inhibited NRF2 expression in a concentration-dependent manner.** (A) PC-9 cells were treated with ADA (5 μM) for the indicated durations, and the expression levels of NRF2 and GAPDH were assessed by Western blot. (B) PC-9 cells were treated with varying concentrations of ADA for 3 hours, followed by Western blot analysis to evaluate NRF2 and GAPDH expression.

**Figure S2**

**
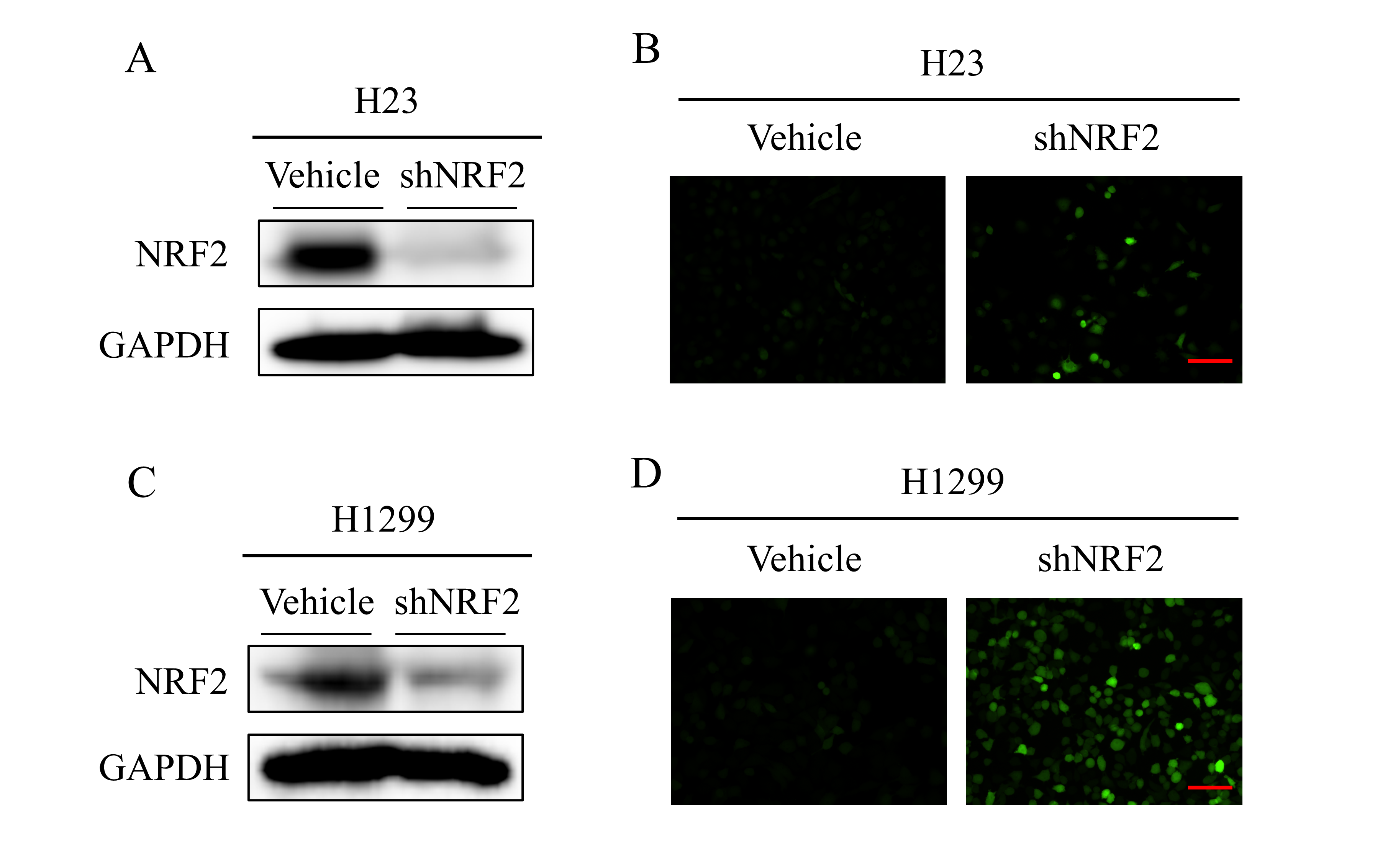
**

**Figure S2: Silencing NRF2 significantly increases ROS level in NSCLC cells.** (A-B) H23 cells were transfected with recombinant lentivirus. The expression levels of NRF2 and GAPDH were subsequently measured by Western blot, and ROS level was assessed using the DCFH-DA probe. (C-D) H1299 cells were transfected with recombinant lentivirus. The expression levels of NRF2 and GAPDH were subsequently measured by Western blot, and ROS level was assessed using the DCFH-DA probe.

**Figure S3**

**
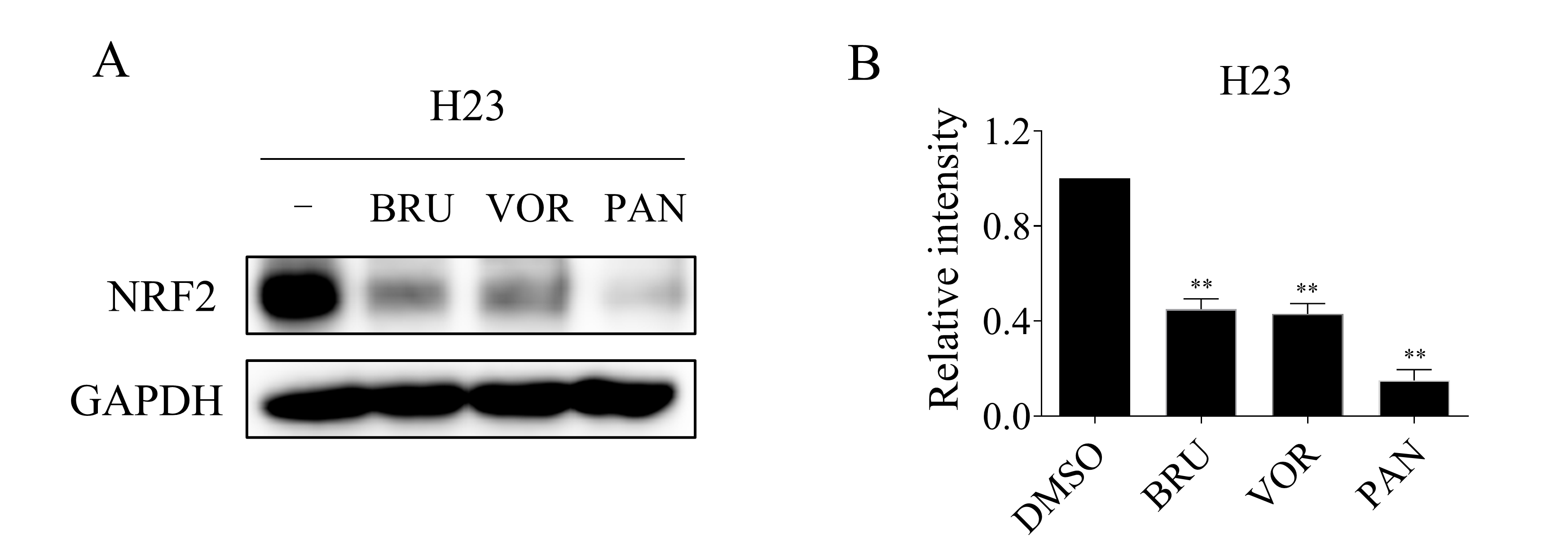
**

**Figure S3: The expression of NRF2 in H23 cells was significantly reduced following treatment with brusatol (BRU), vorinostat (VOR), or panobinostat (PAN).** (A-B) H23 cells were treated with BRU, VOR, or PAN for 3 hours, followed by Western blot analysis to evaluate NRF2 and GAPDH expression. ***p* < 0.01, as determined by One-way ANOVA.
