# Supplementary material for: Panobinostat potentiates adagrasib-induced cell death by triggering autophagy in human non-small cell lung cancer
Source: Cell Death Discov. 2025 Aug 1;11:360. doi: 10.1038/s41420-025-02657-9 (PMC12316882; doi:10.1038/s41420-025-02657-9)

Figure 1

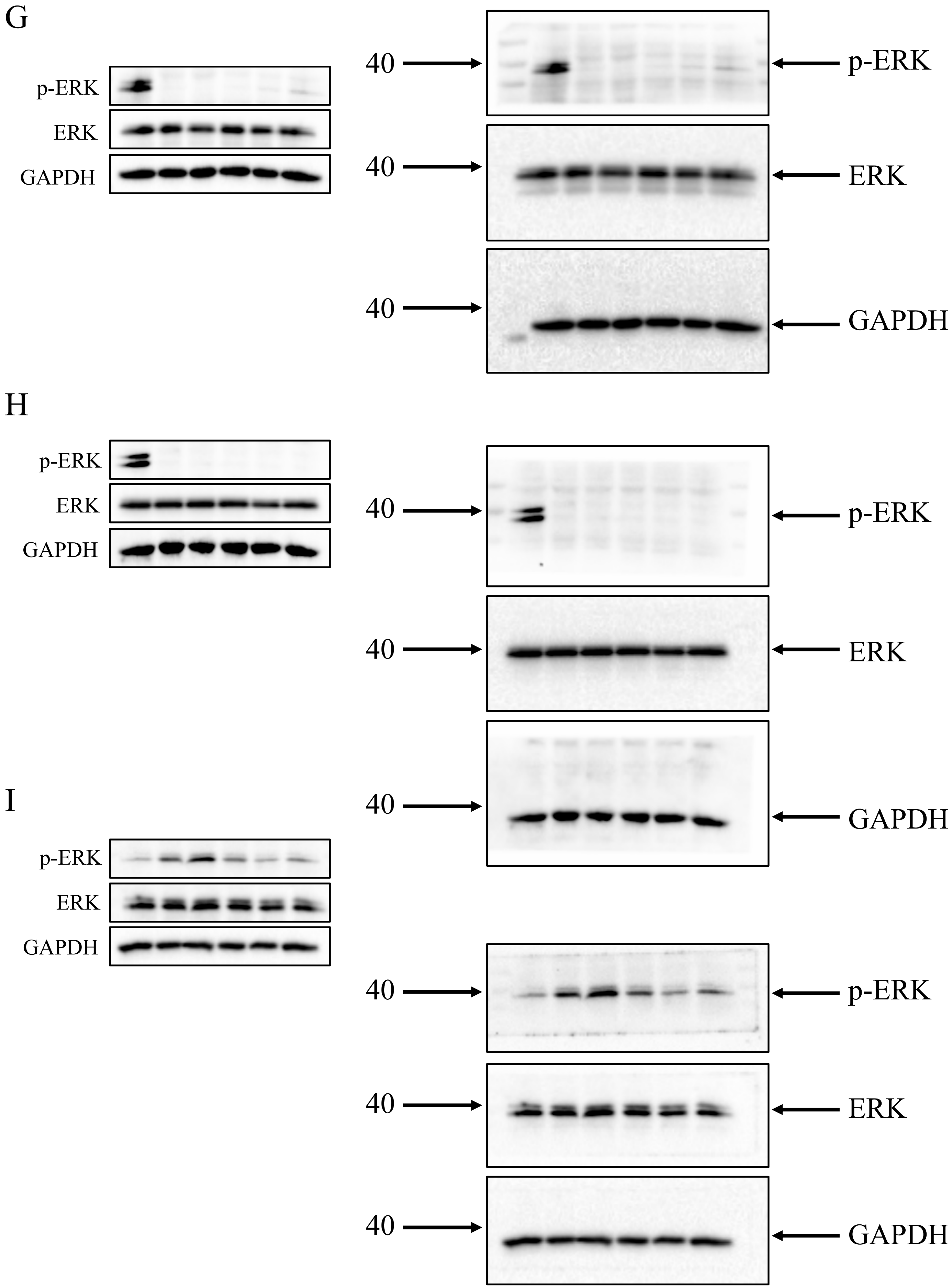
G
p-ERK
ERK
GAPDH
40
40
40
p-ERK
ERK
GAPDH
H
p-ERK
ERK
GAPDH
40
40
40
p-ERK
ERK
GAPDH
I
p-ERK
ERK
GAPDH
40
40
40
p-ERK
ERK
GAPDH

Figure 1

J

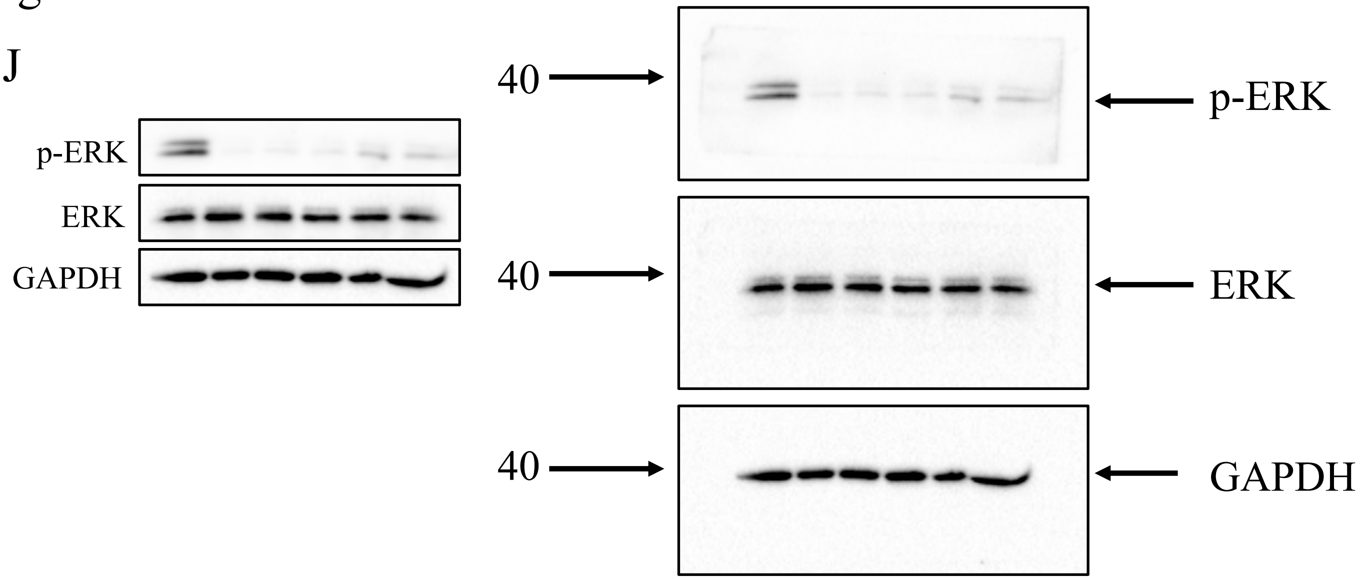

p-ERK
ERK
GAPDH
40
40
40
p-ERK
ERK
GAPDH

K

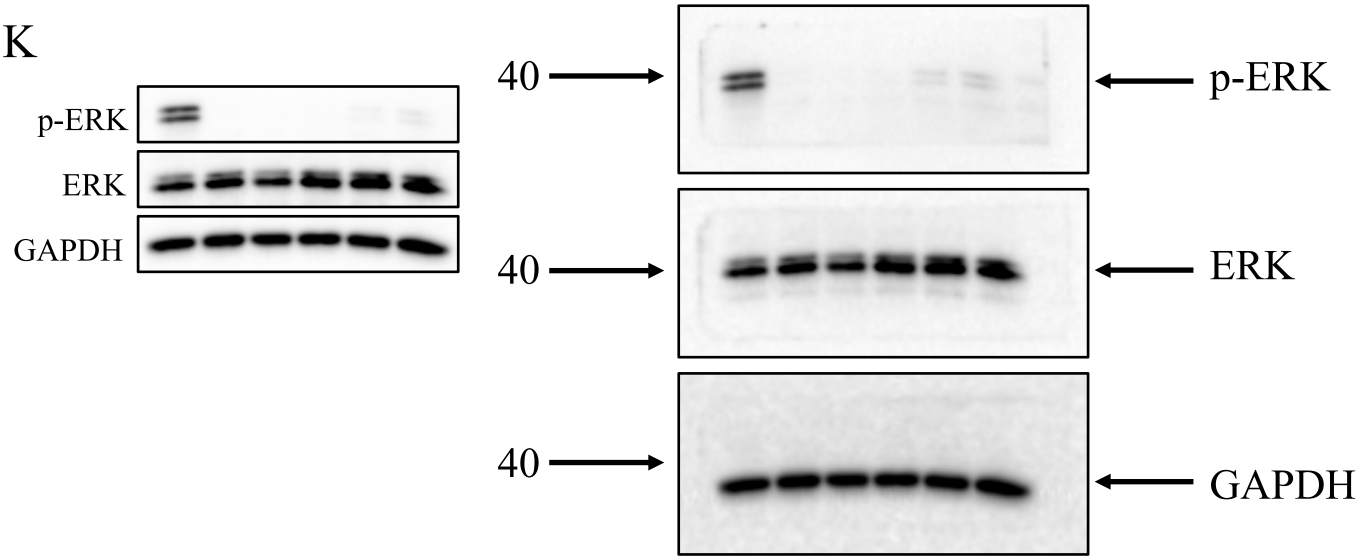

p-ERK
ERK
GAPDH
40
40
40
p-ERK
ERK
GAPDH

L

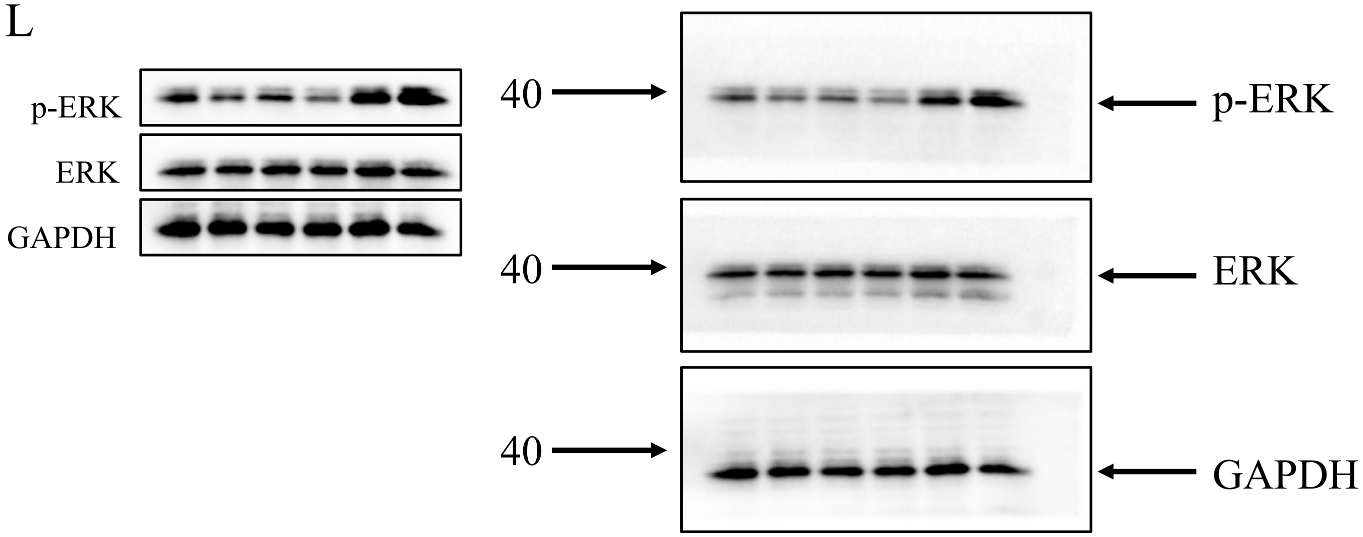

p-ERK
ERK
GAPDH
40
40
40
p-ERK
ERK
GAPDH

Figure 2

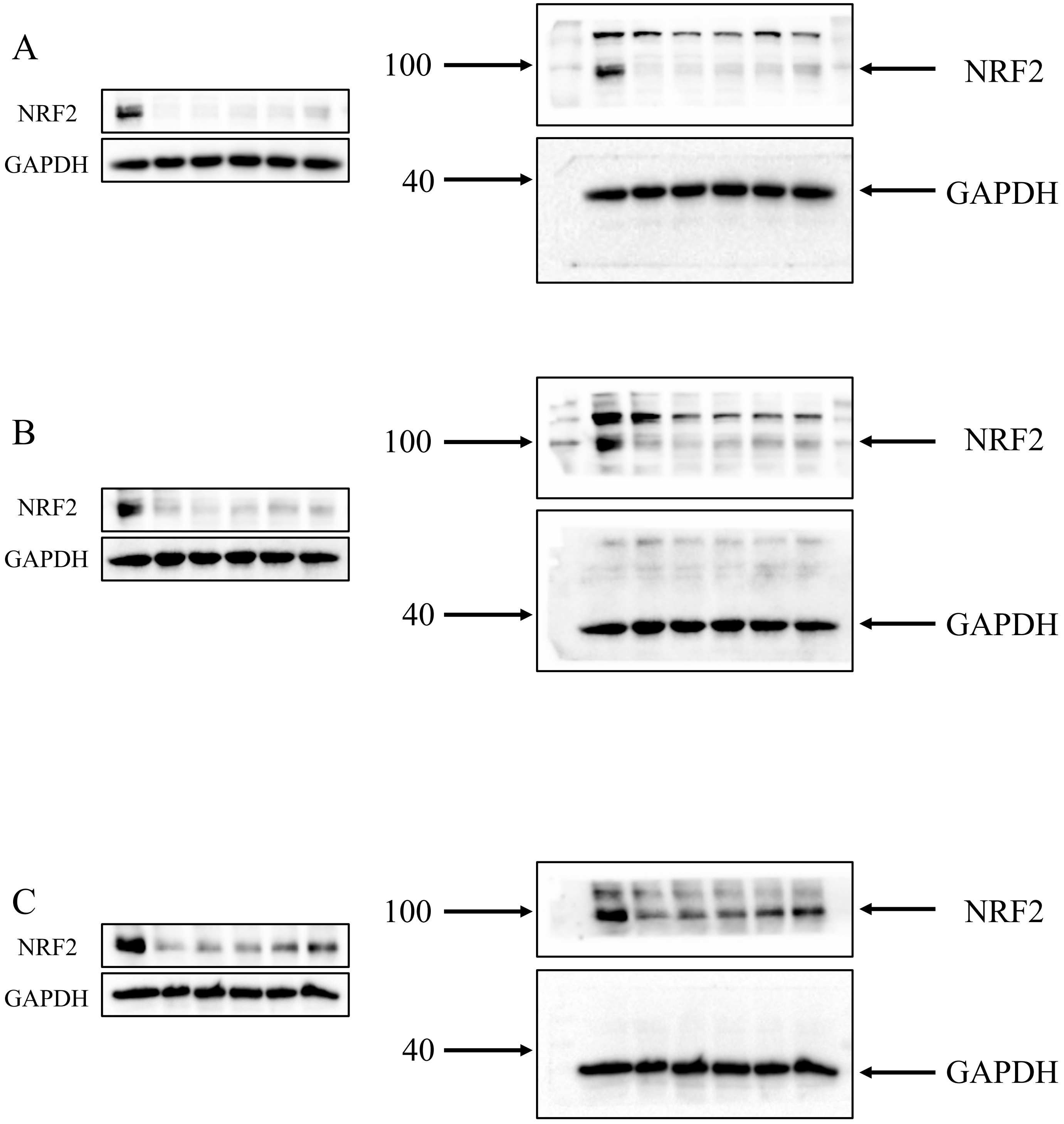
A
NRF2
GAPDH
100
NRF2
40
GAPDH
B
NRF2
GAPDH
100
NRF2
40
GAPDH
C
NRF2
GAPDH
100
NRF2
40
GAPDH

Figure 2

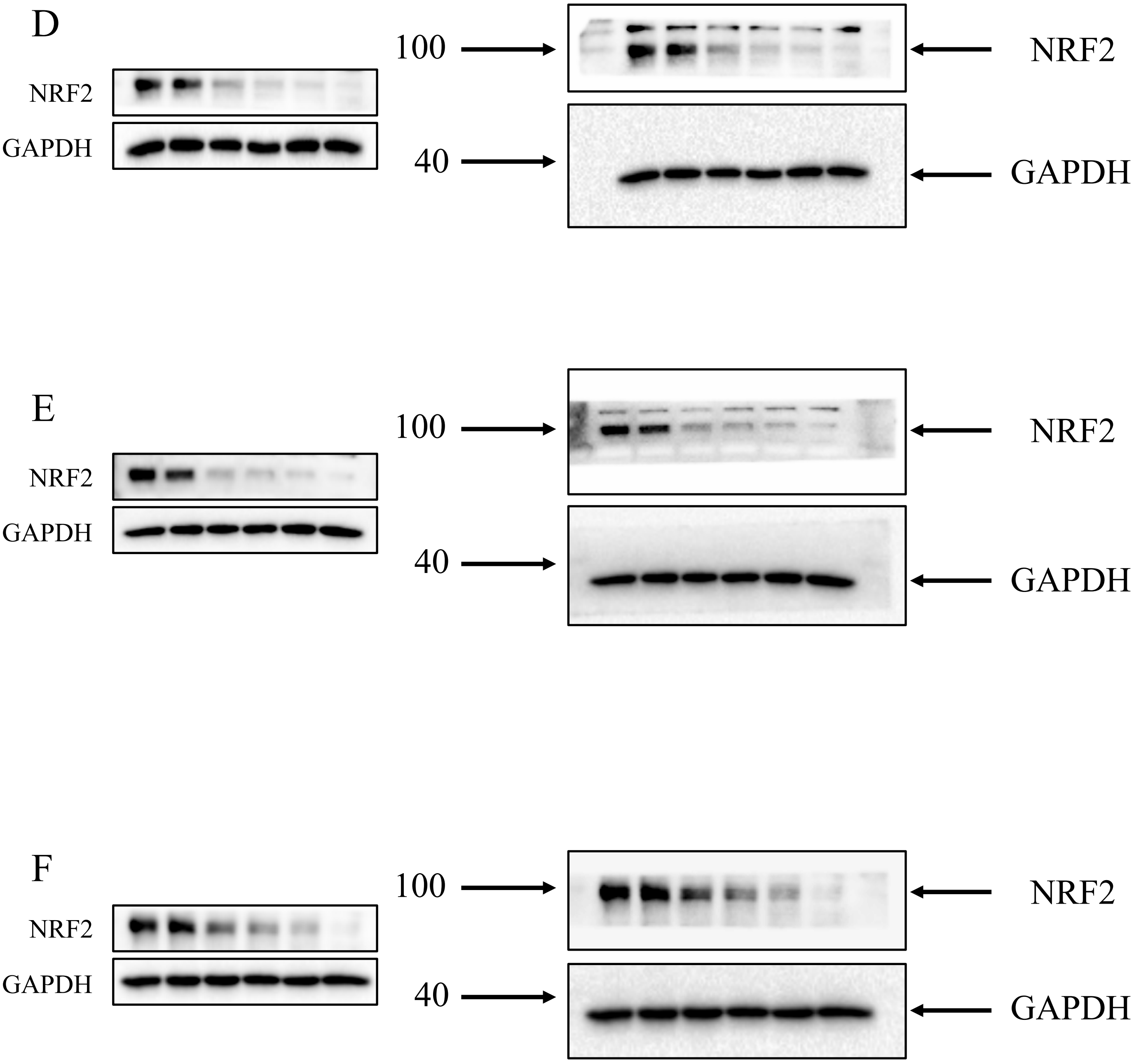

D
NRF2
GAPDH
100
40
NRF2
GAPDH
E
NRF2
GAPDH
100
40
NRF2
GAPDH
F
NRF2
GAPDH
100
40
NRF2
GAPDH

Figure 2

G

H23

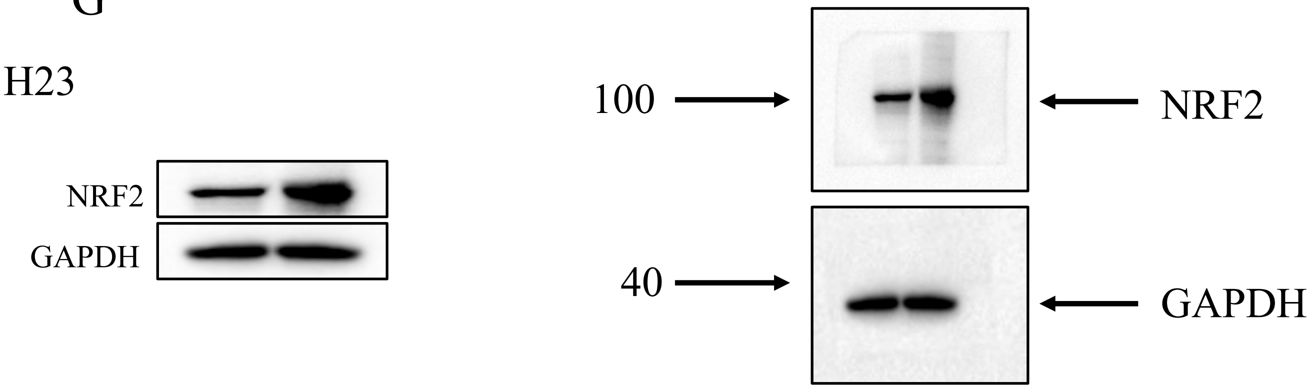

H1299

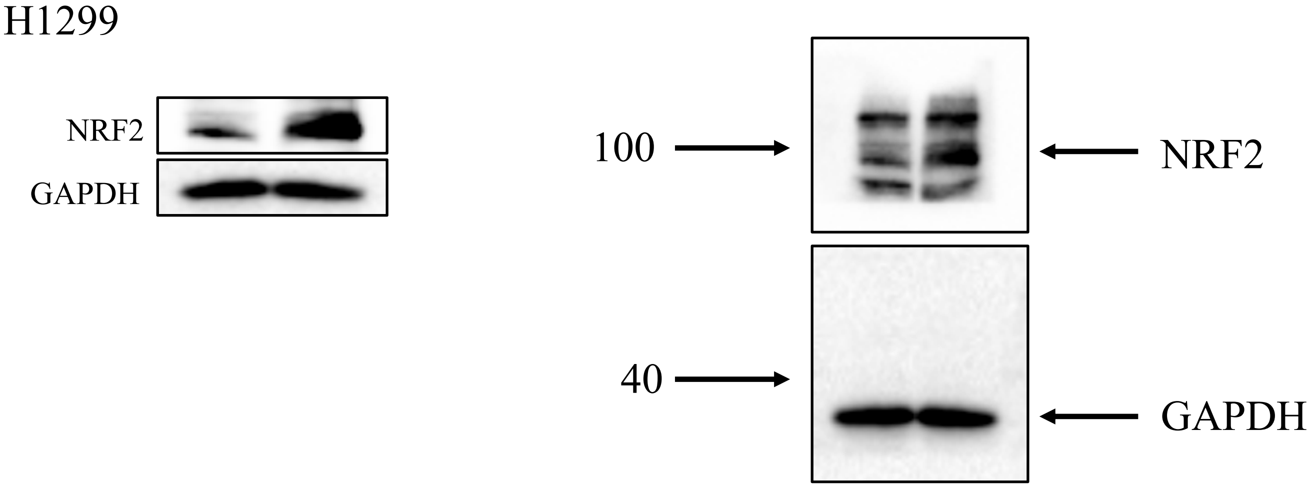

J

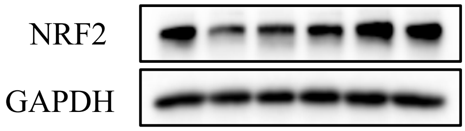

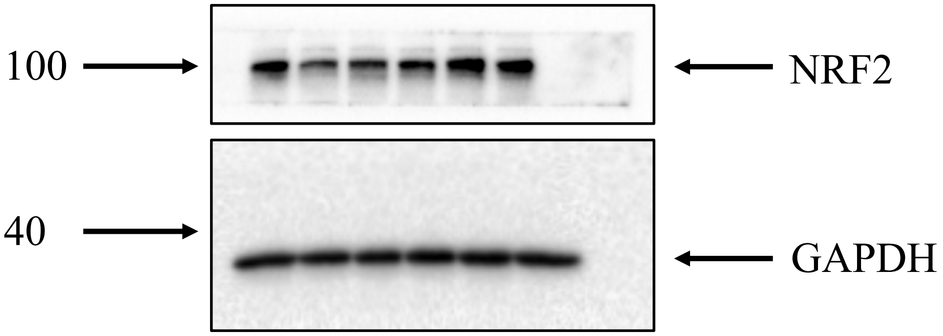

Figure 3

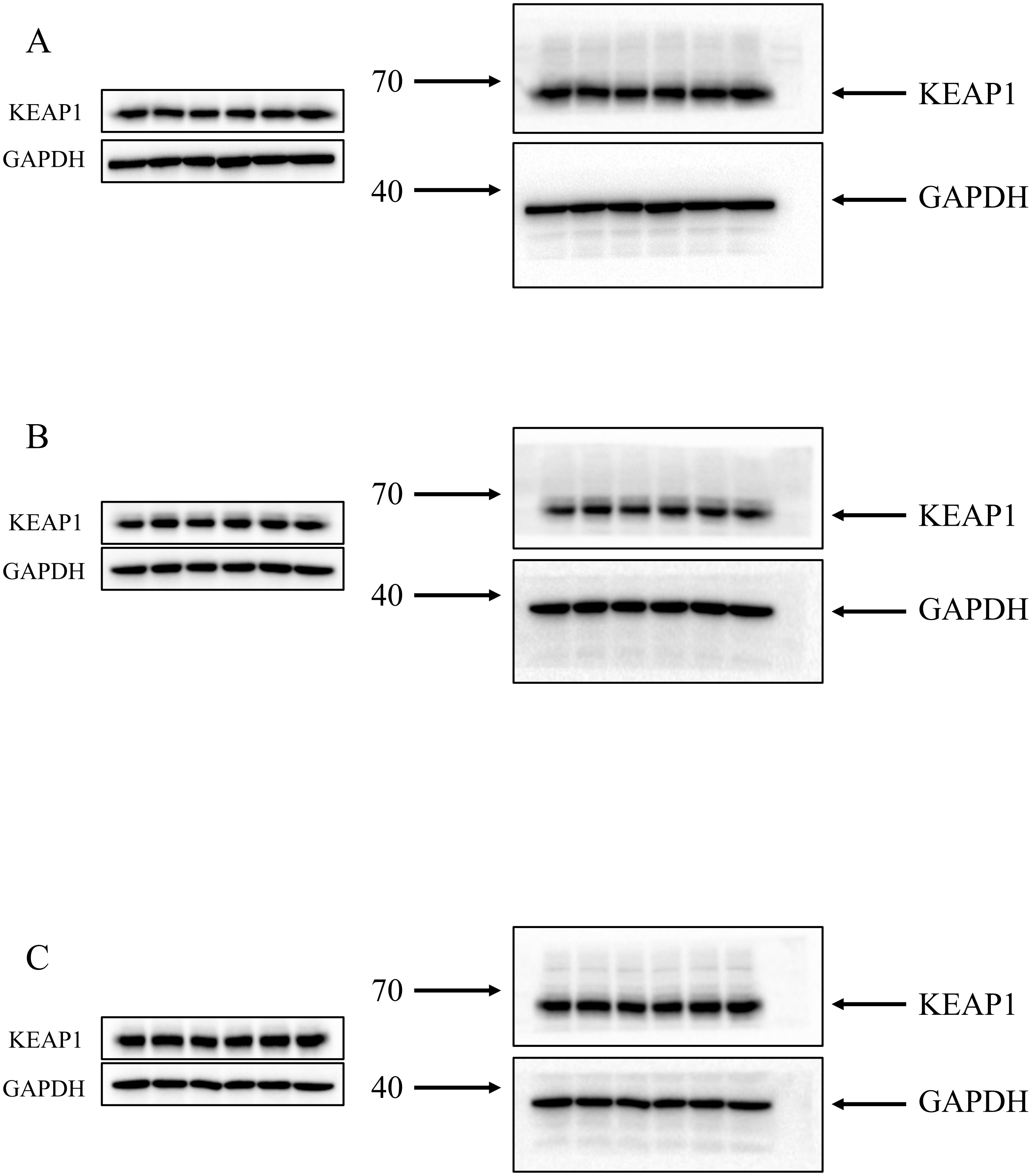
A
KEAP1
GAPDH
70
40
KEAP1
GAPDH
B
KEAP1
GAPDH
70
40
KEAP1
GAPDH
C
KEAP1
GAPDH
70
40
KEAP1
GAPDH

Figure 3

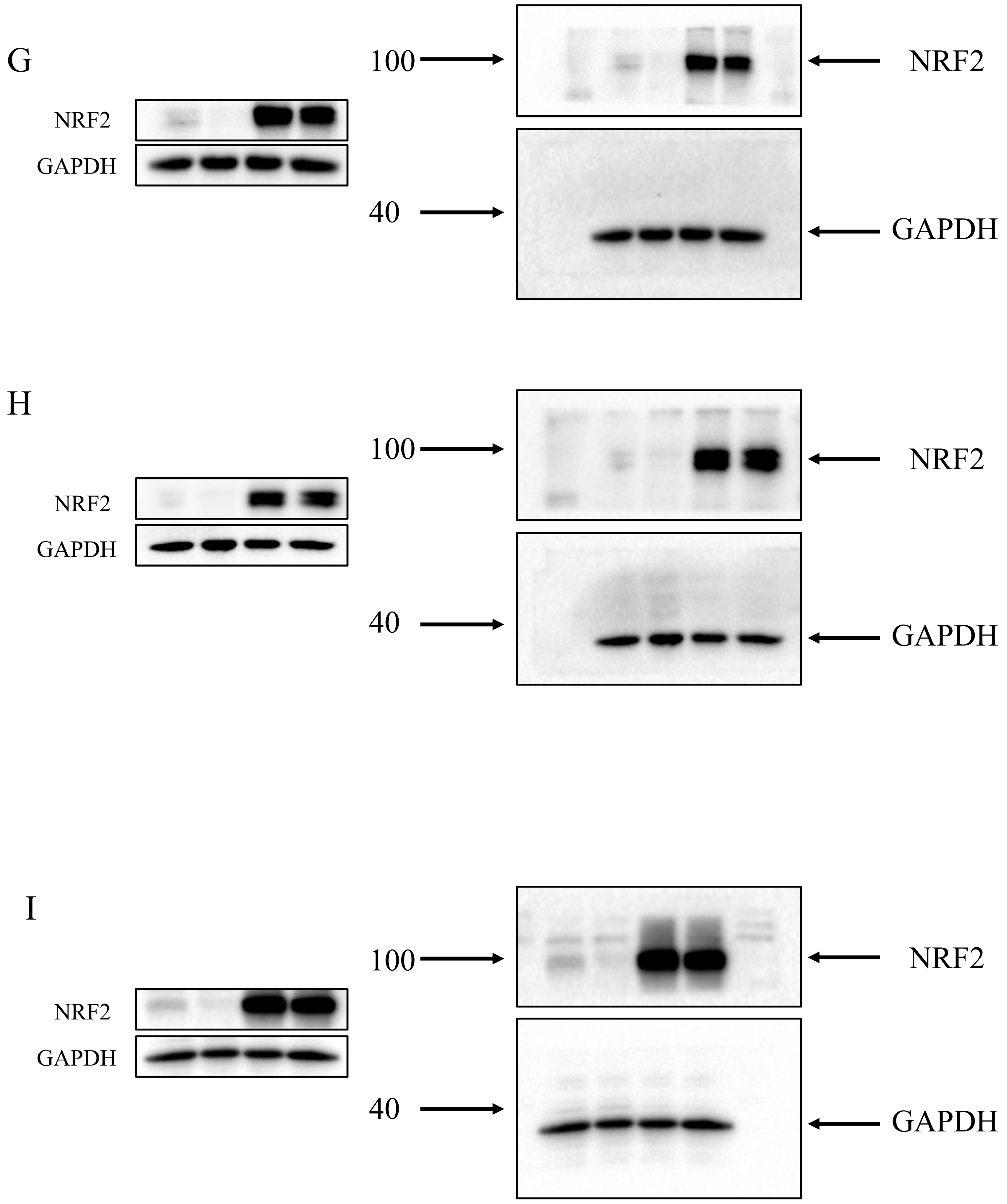
G
NRF2
GAPDH
100
40
NRF2
GAPDH
H
NRF2
GAPDH
100
40
NRF2
GAPDH
I
NRF2
GAPDH
100
40
NRF2
GAPDH

Figure 3

J

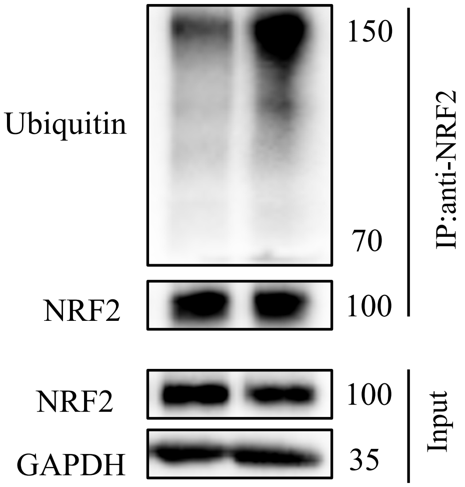

Ubiquitin
150
70
IP:anti-NRF2
NRF2
100
NRF2
100
Input
GAPDH
35

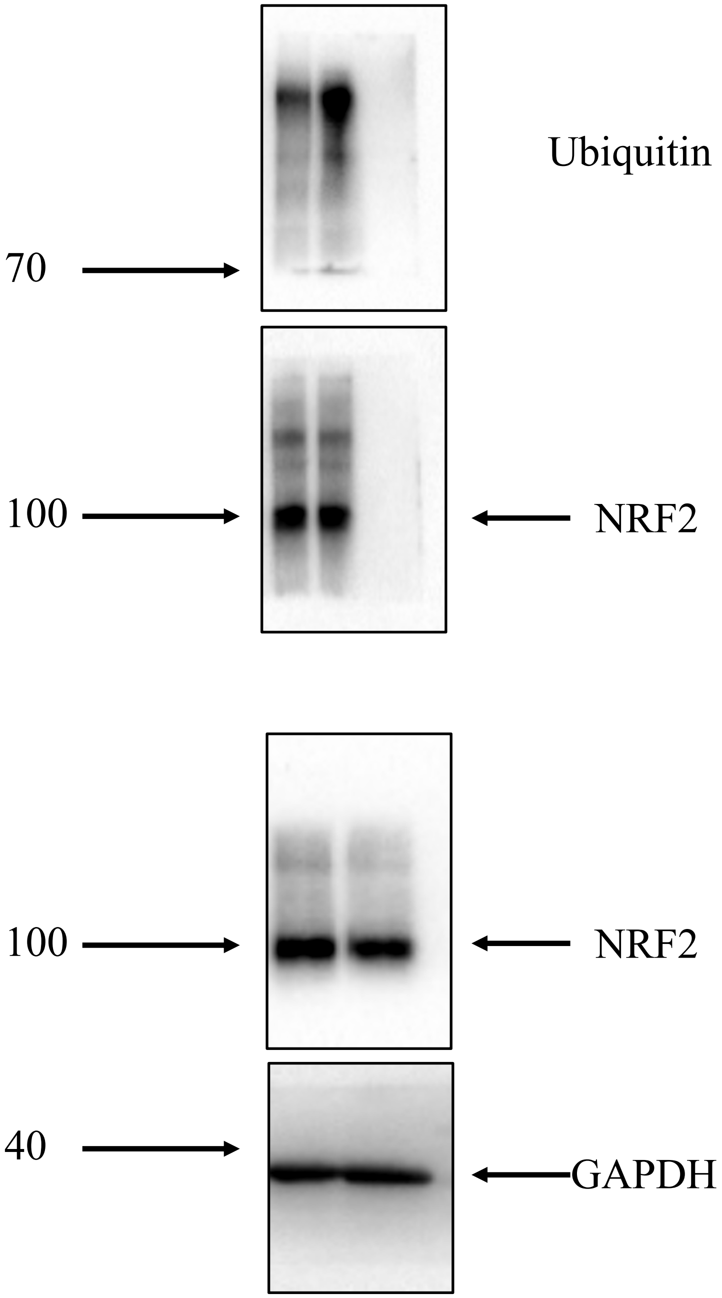

Ubiquitin
70
100
NRF2
100
NRF2
40
GAPDH

Figure 3

K

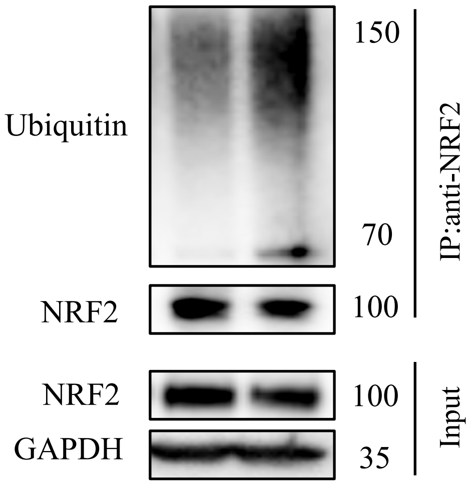

Ubiquitin
150
70
NRF2
100
IP:anti-NRF2
NRF2
100
GAPDH
35
Input

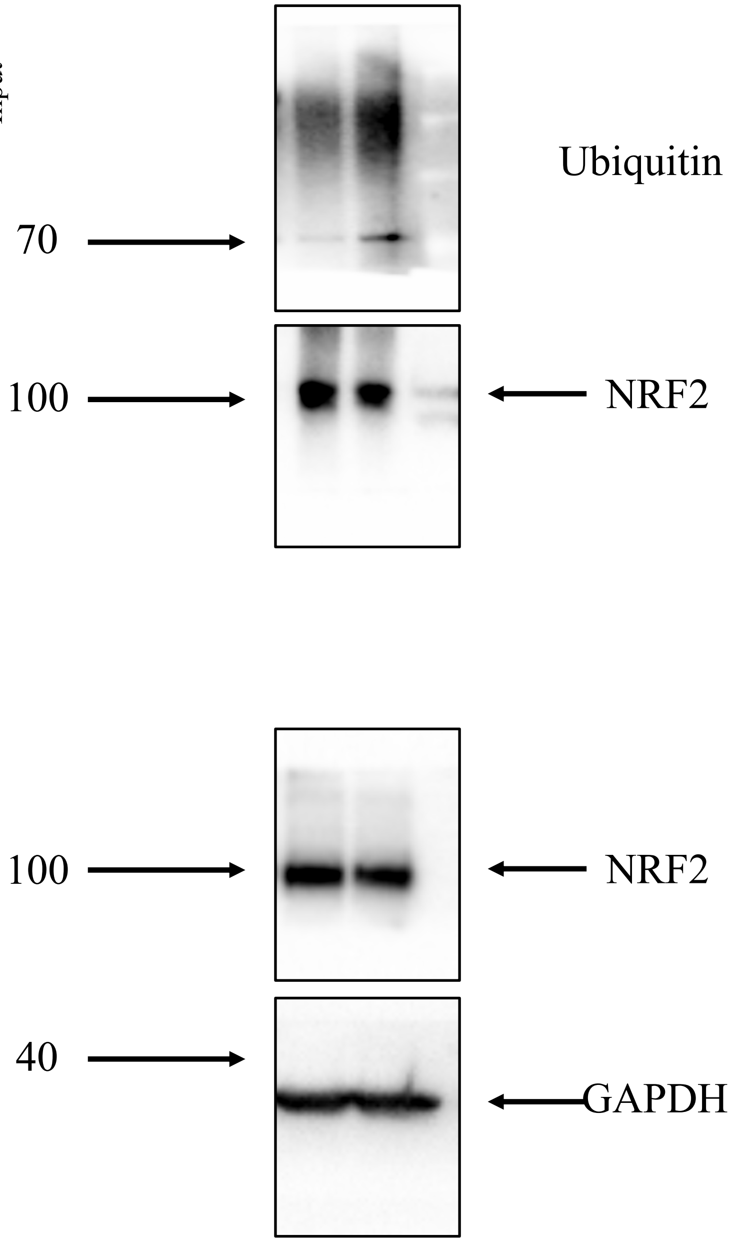

70
Ubiquitin
100
NRF2
100
NRF2
40
GAPDH

Figure 3

L

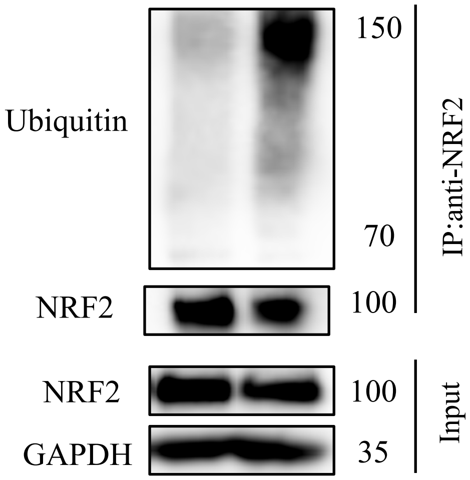

Ubiquitin
150
70
IP:anti-NRF2
NRF2
100
NRF2
100
Input
GAPDH
35

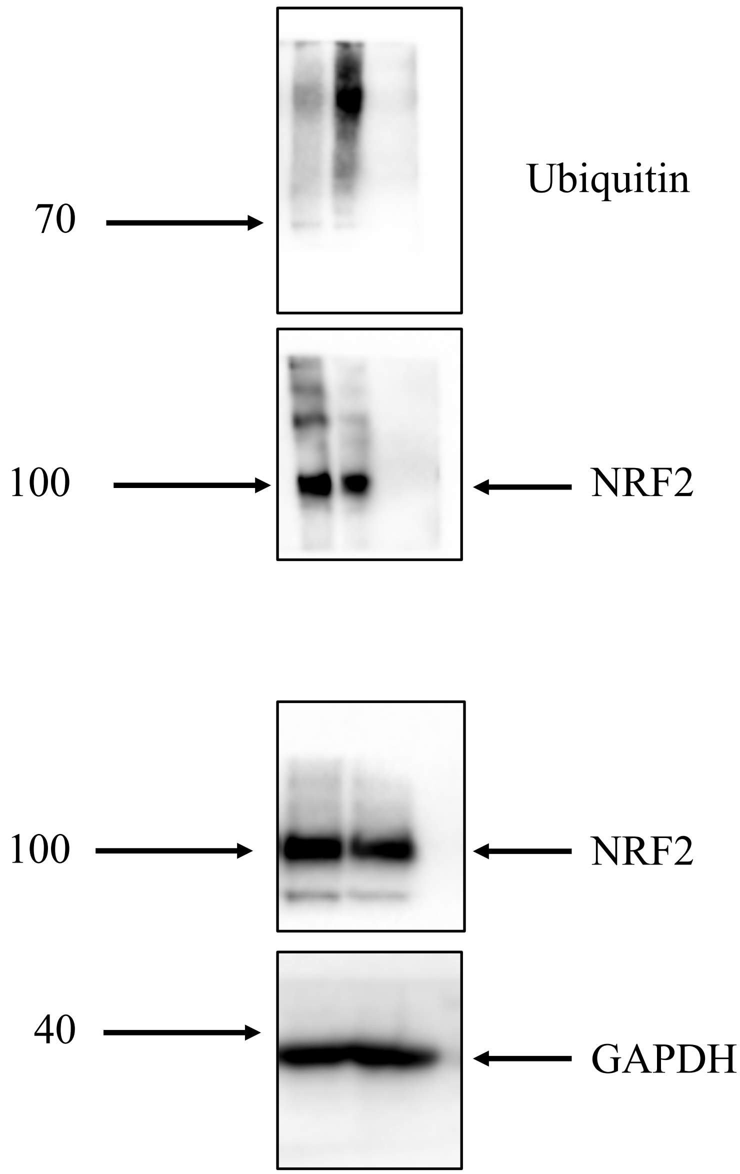

70
Ubiquitin
100
NRF2
100
NRF2
40
GAPDH

Figure 5

A

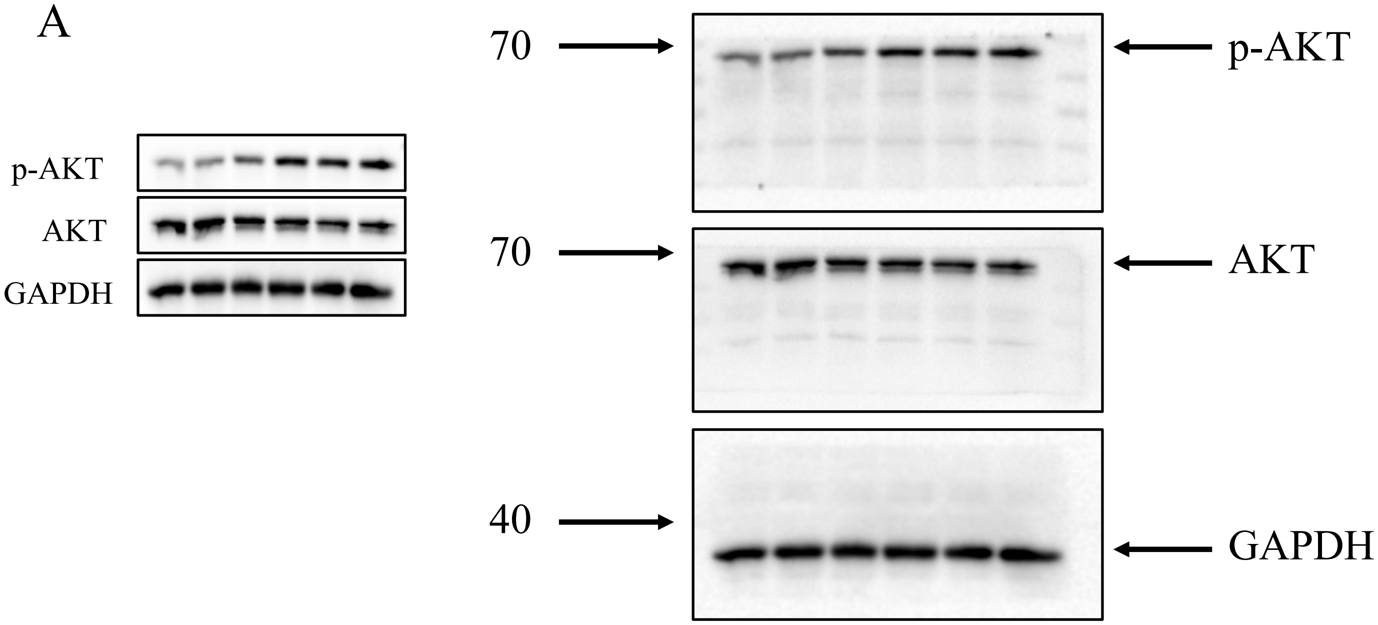
p-AKT
AKT
GAPDH
70
70
40
p-AKT
AKT
GAPDH

B

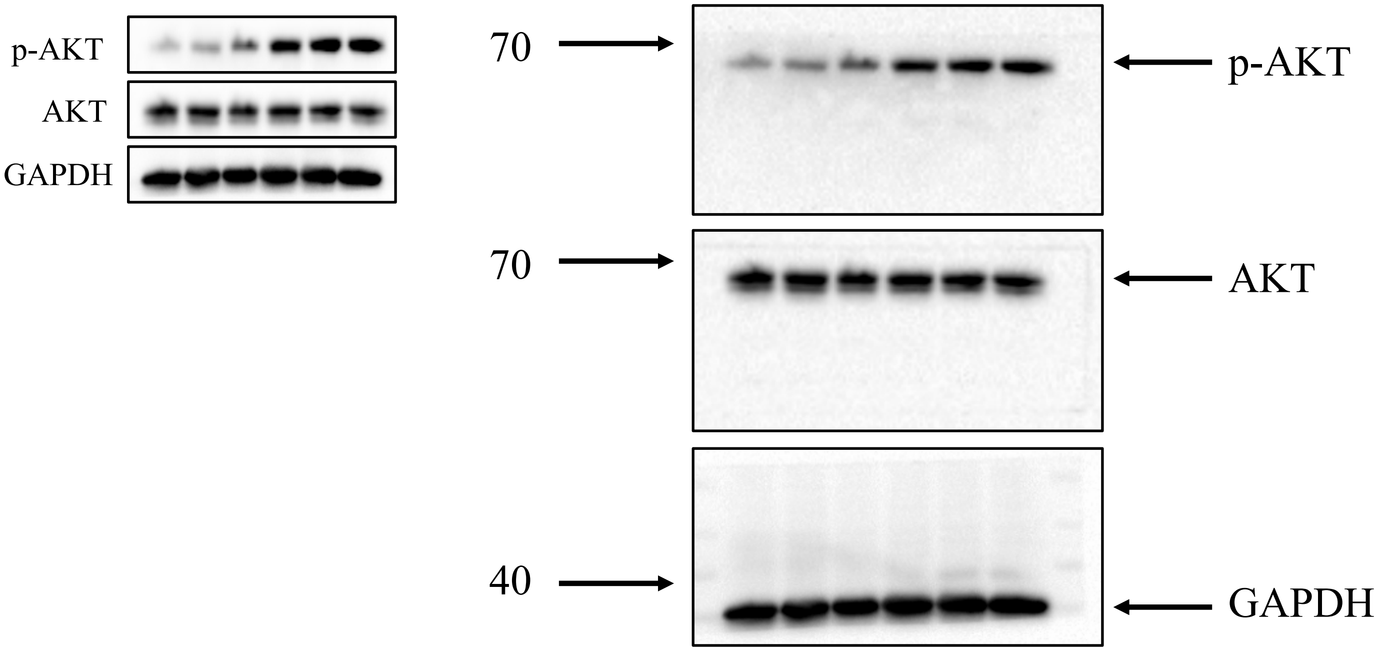
p-AKT
AKT
GAPDH
70
70
40
p-AKT
AKT
GAPDH

Figure 5

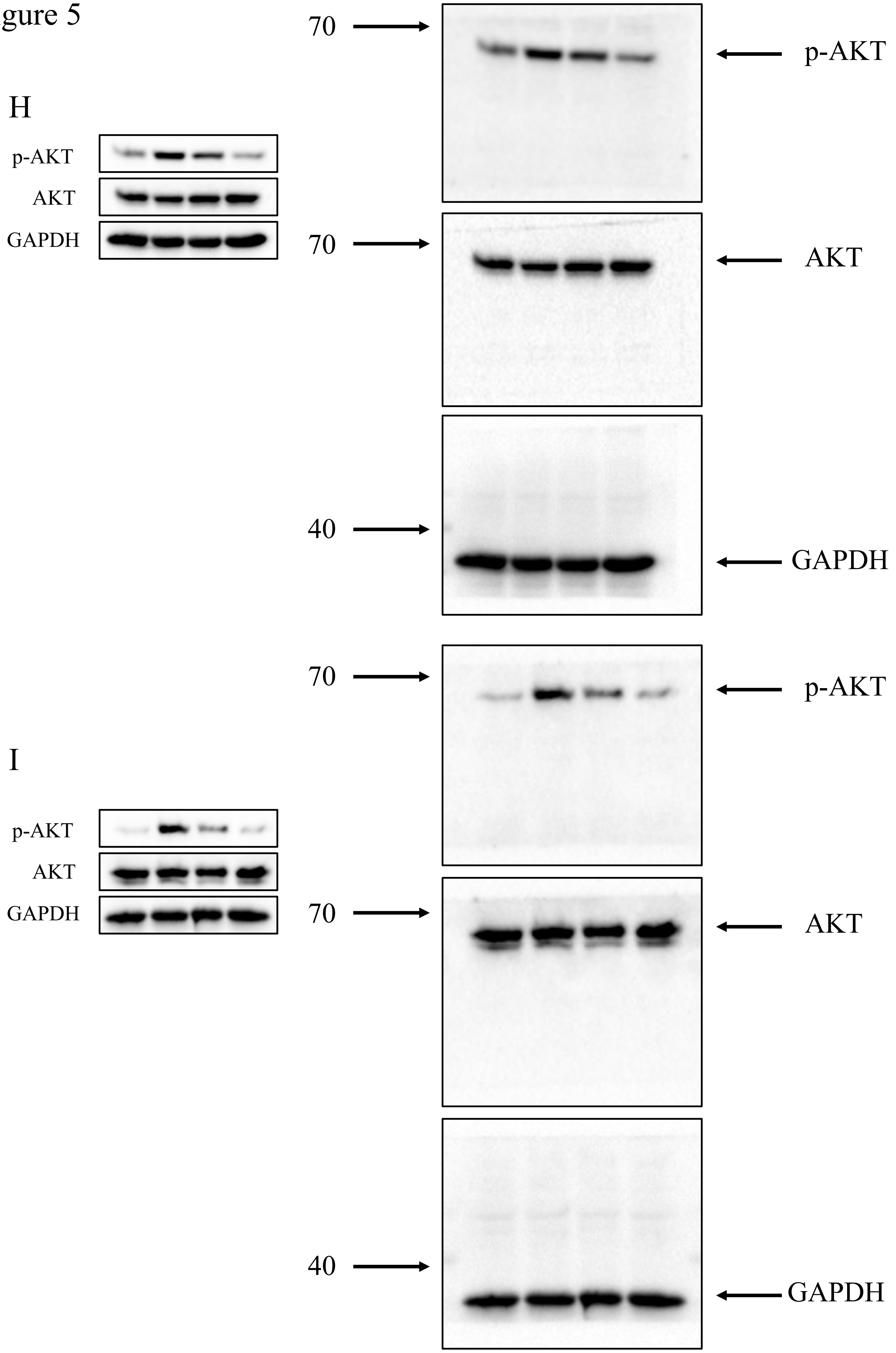
H
p-AKT
AKT
GAPDH
70
70
40
p-AKT
AKT
GAPDH
I
p-AKT
AKT
GAPDH
70
70
40
p-AKT
AKT
GAPDH

Figure 6

A

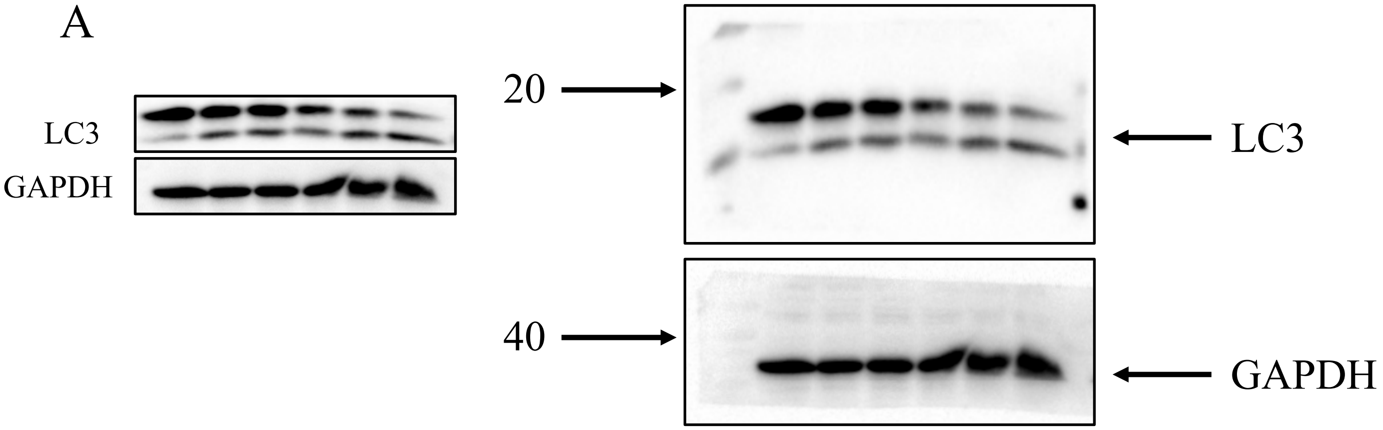
LC3
GAPDH
20
LC3
40
GAPDH

B

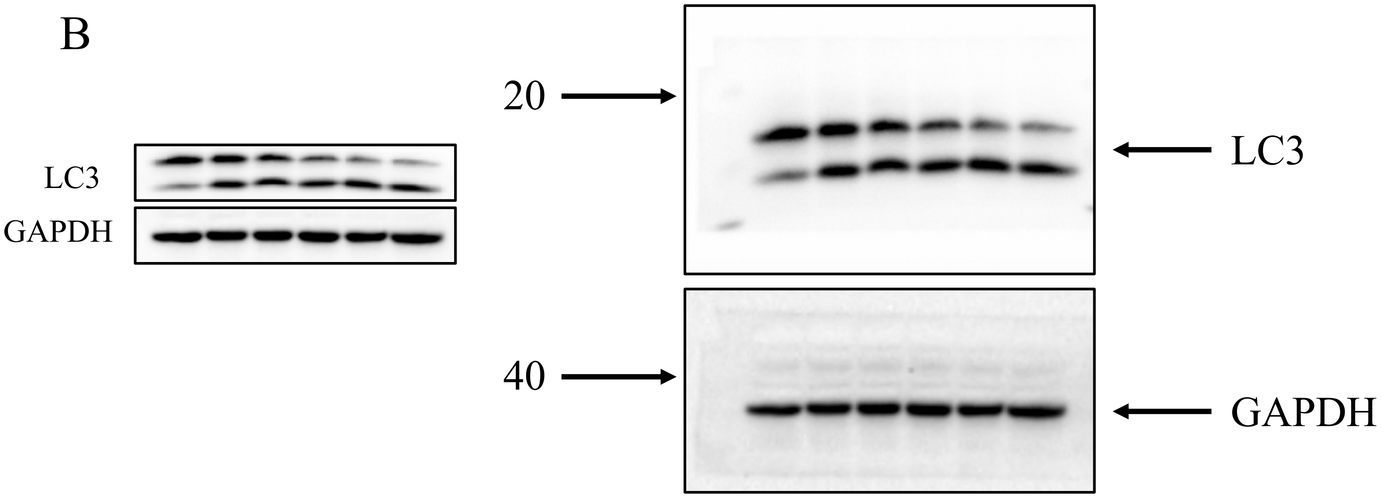
LC3
GAPDH
20
LC3
40
GAPDH

C

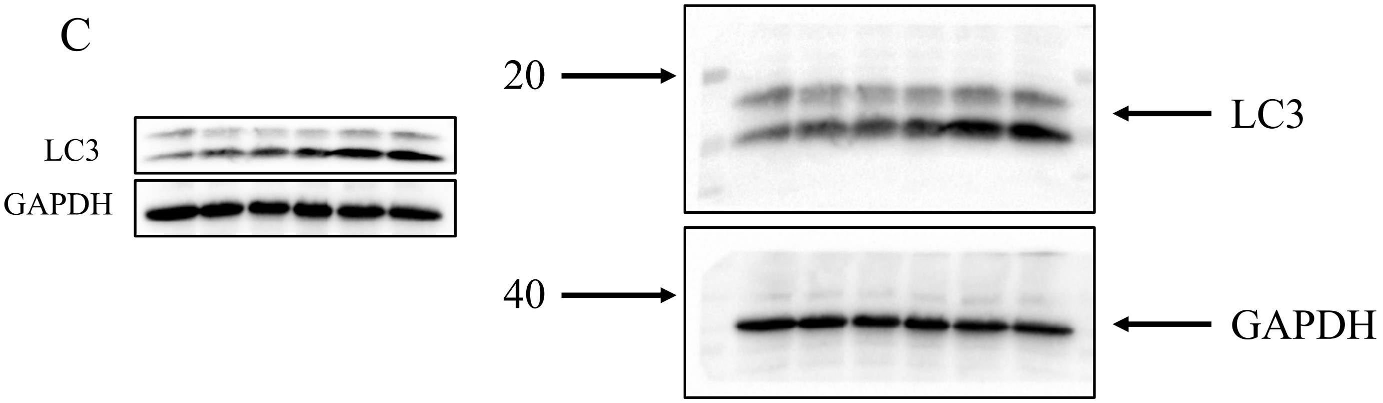
LC3
GAPDH
20
LC3
40
GAPDH

Figure 6

D

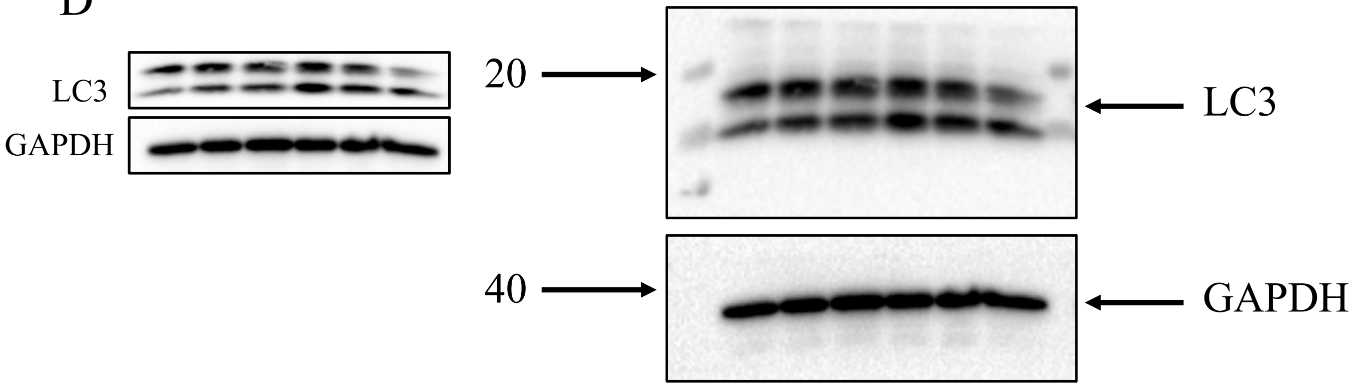
LC3
GAPDH
20
40
LC3
GAPDH

E

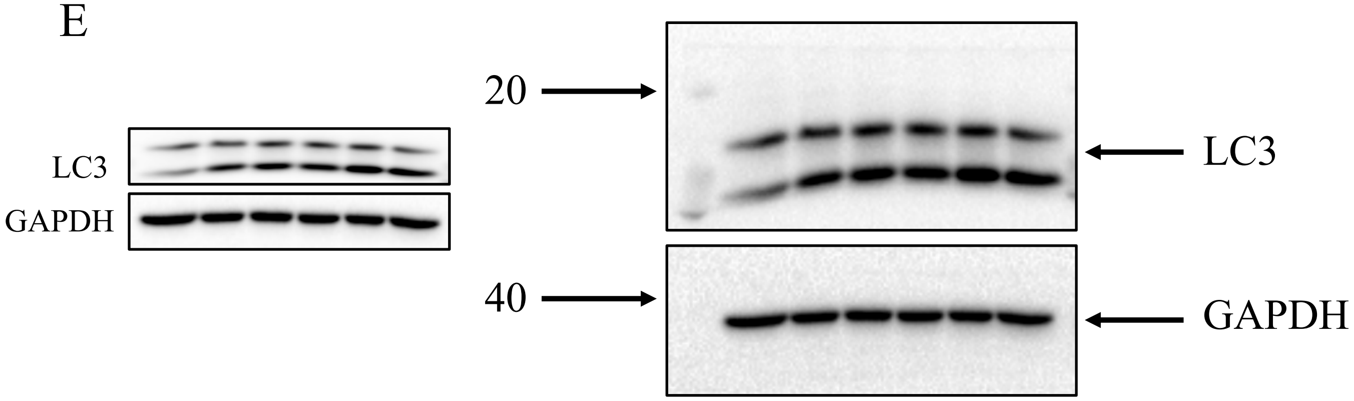
LC3
GAPDH
20
40
LC3
GAPDH

F

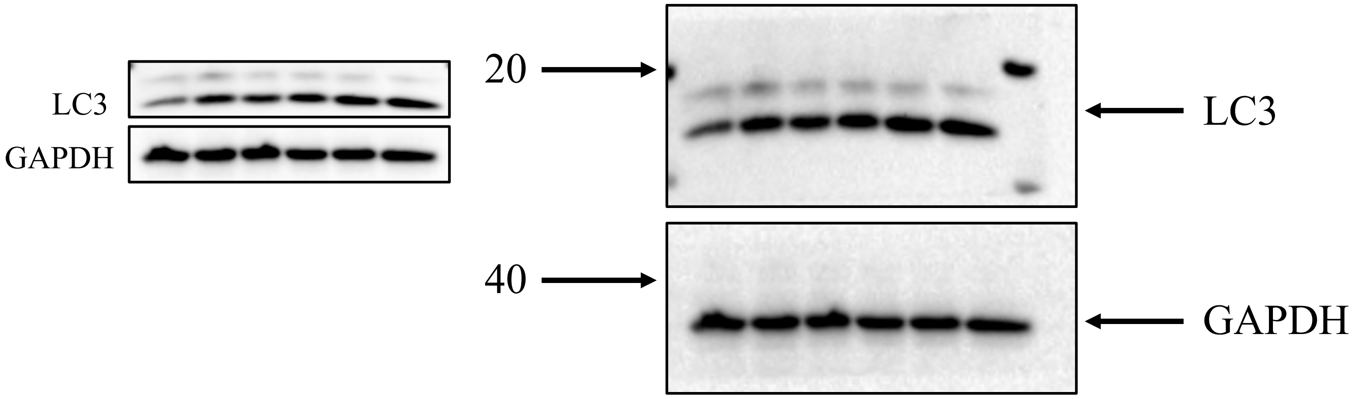
LC3
GAPDH
20
40
LC3
GAPDH

Figure 7

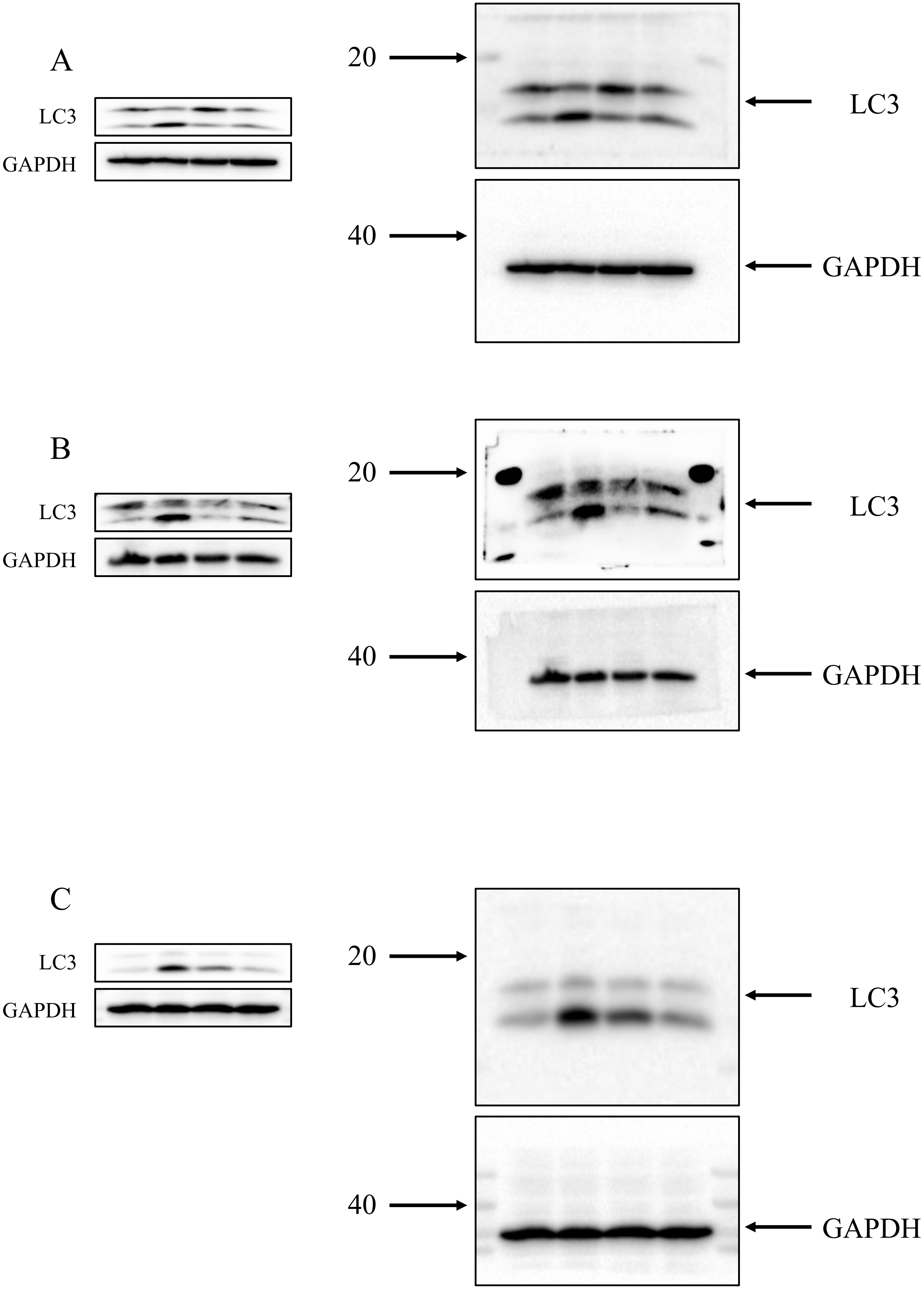
A
LC3
GAPDH
20
40
LC3
GAPDH
B
LC3
GAPDH
20
40
LC3
GAPDH
C
LC3
GAPDH
20
40
LC3
GAPDH

Figure 7

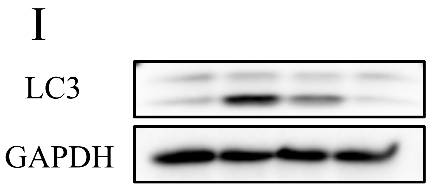

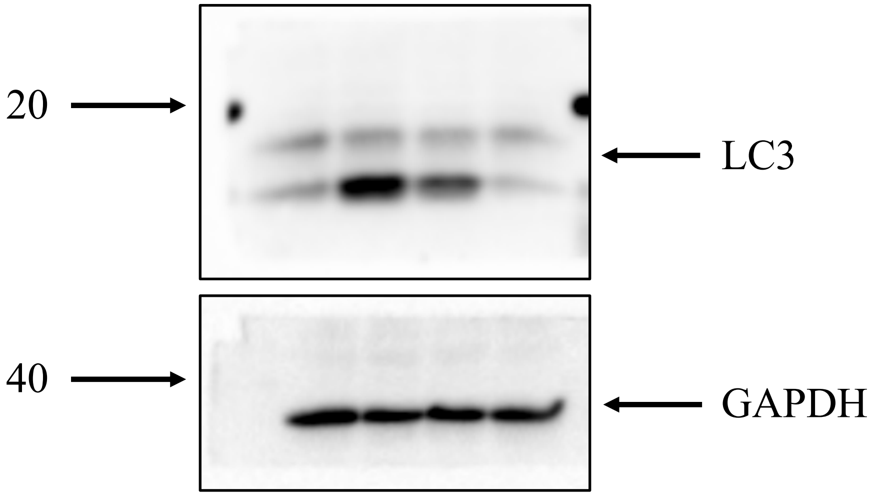

Figure 8

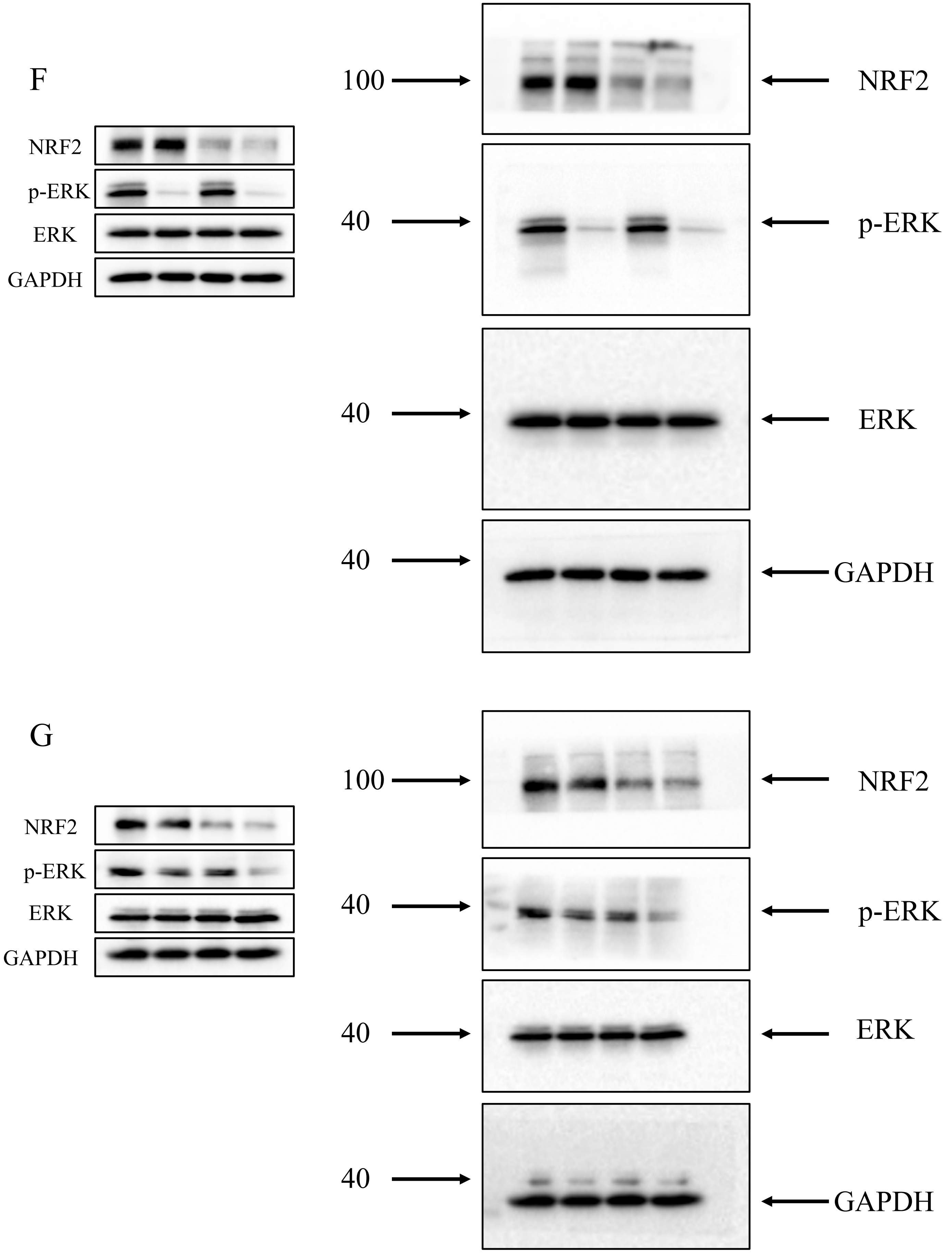

F
NRF2
p-ERK
ERK
GAPDH
100
40
40
40
NRF2
p-ERK
ERK
GAPDH
G
NRF2
p-ERK
ERK
GAPDH
100
40
40
40
NRF2
p-ERK
ERK
GAPDH

Figure 8

H

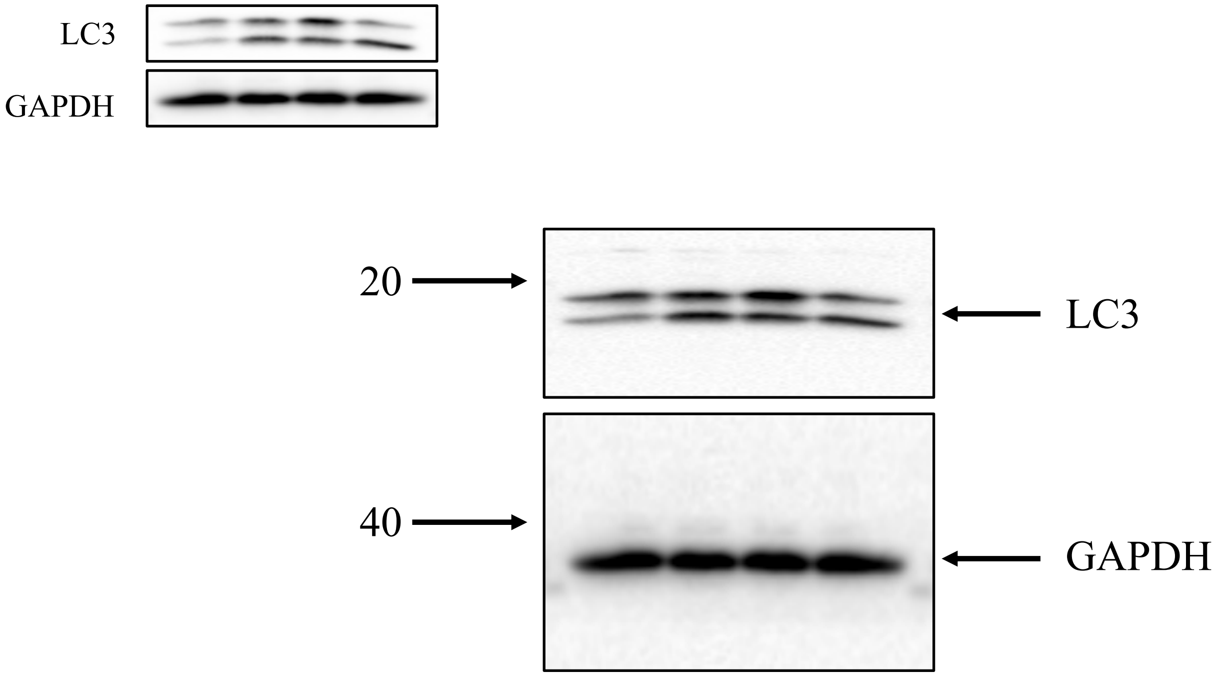

I

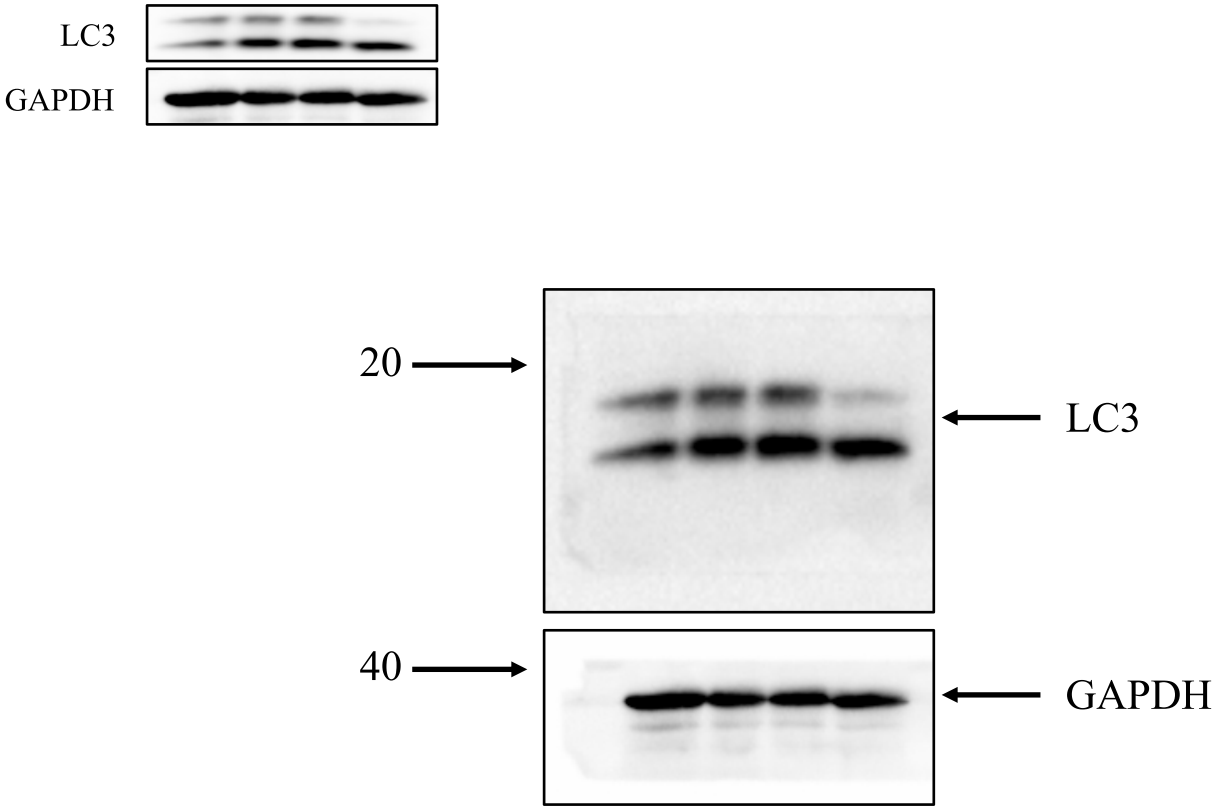

Figure 9

A

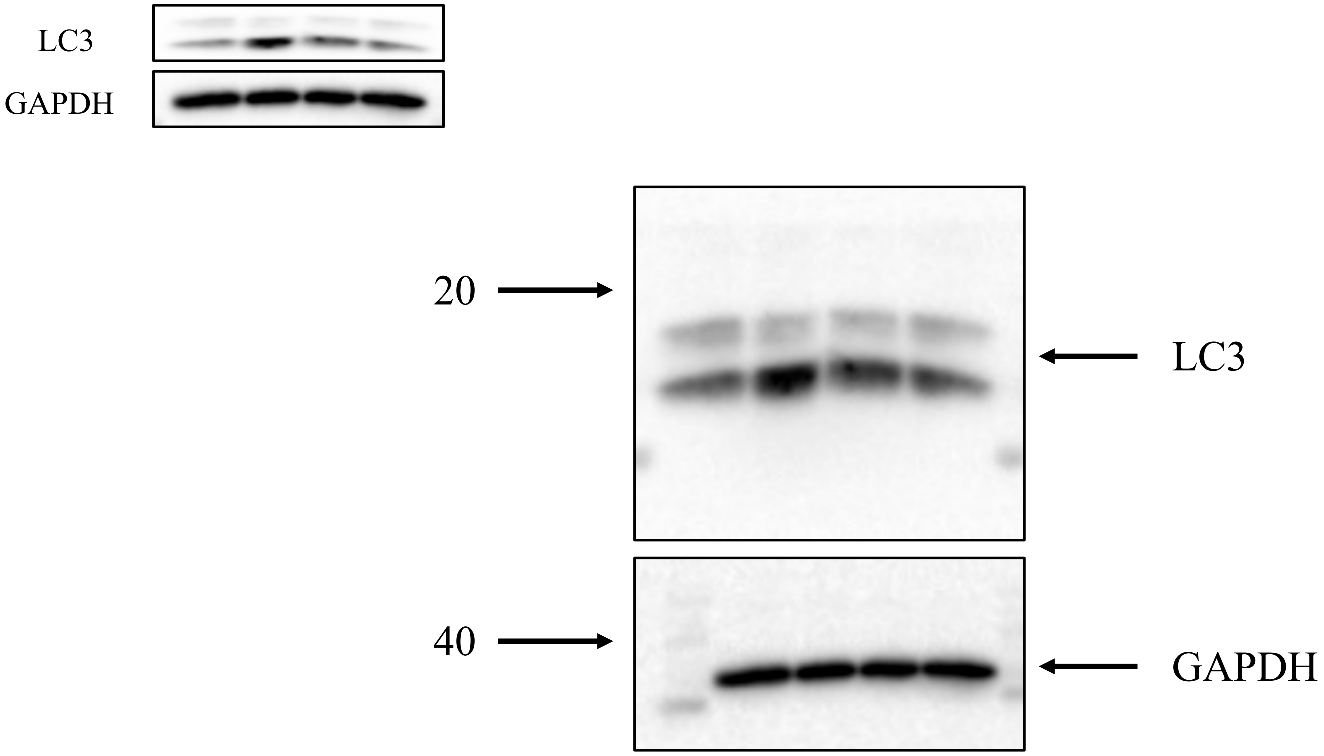

F

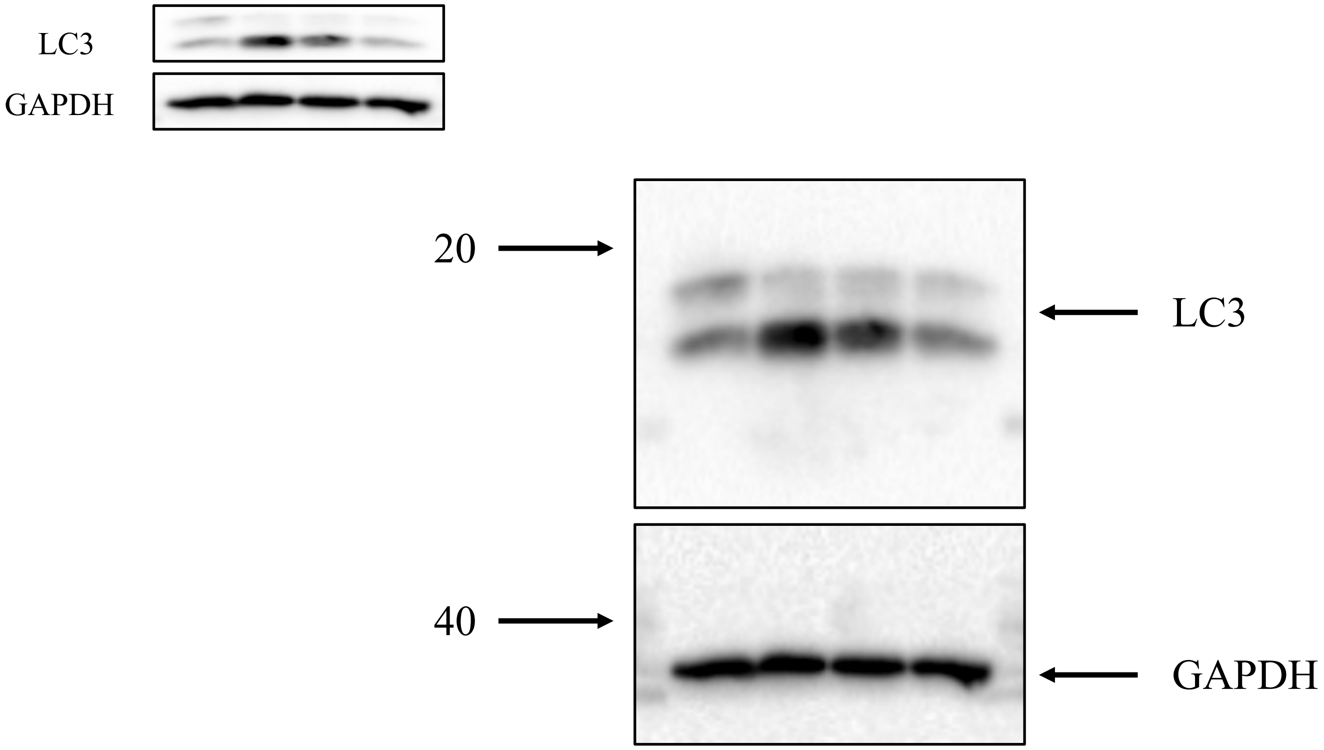

Figure 10

G

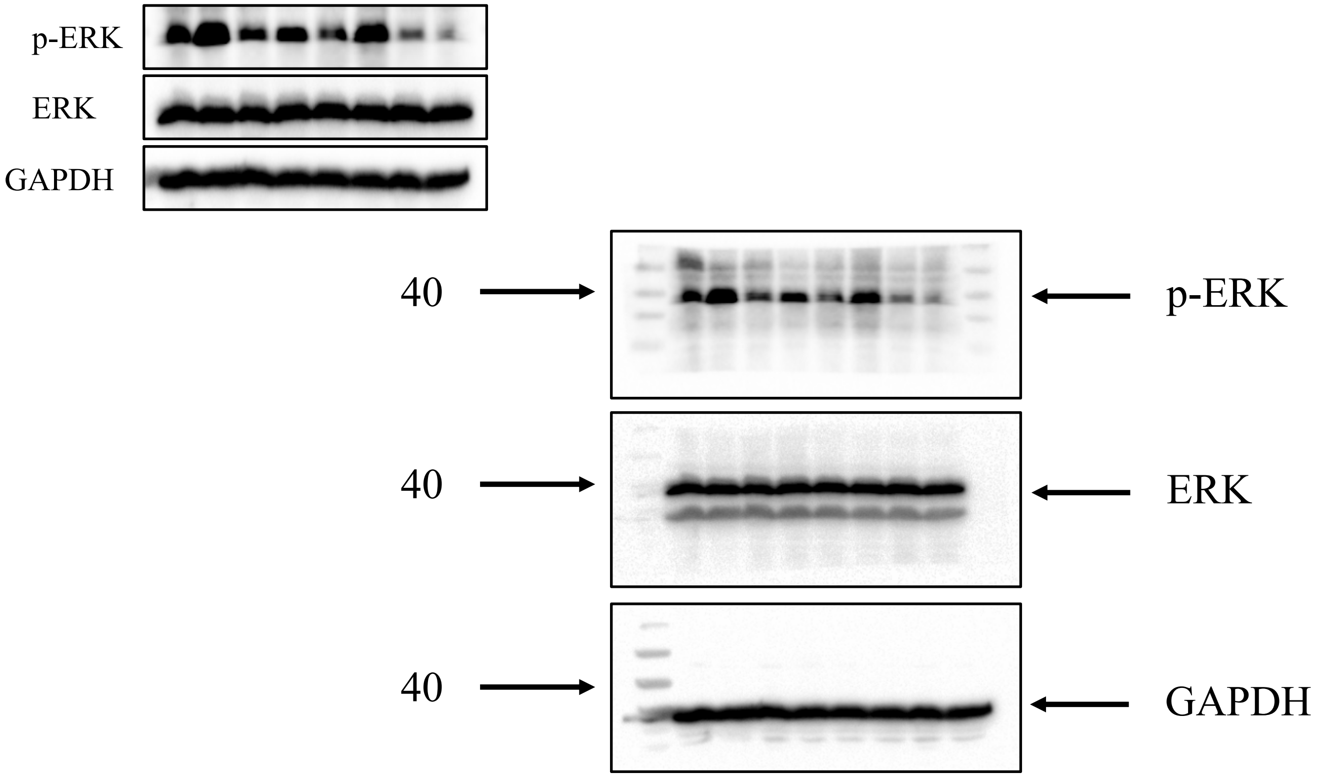
p-ERK
ERK
GAPDH
40
40
40
p-ERK
ERK
GAPDH

H

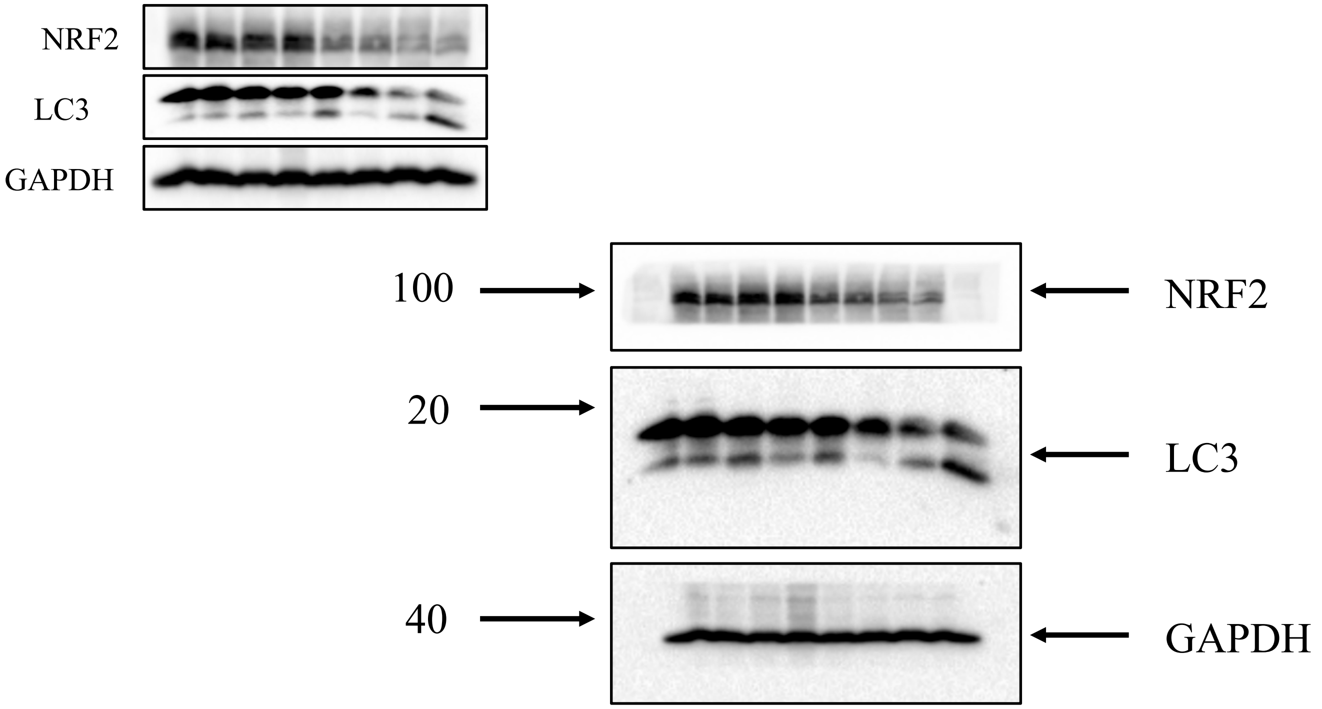
NRF2
LC3
GAPDH
100
20
40
NRF2
LC3
GAPDH

Figure S1

A

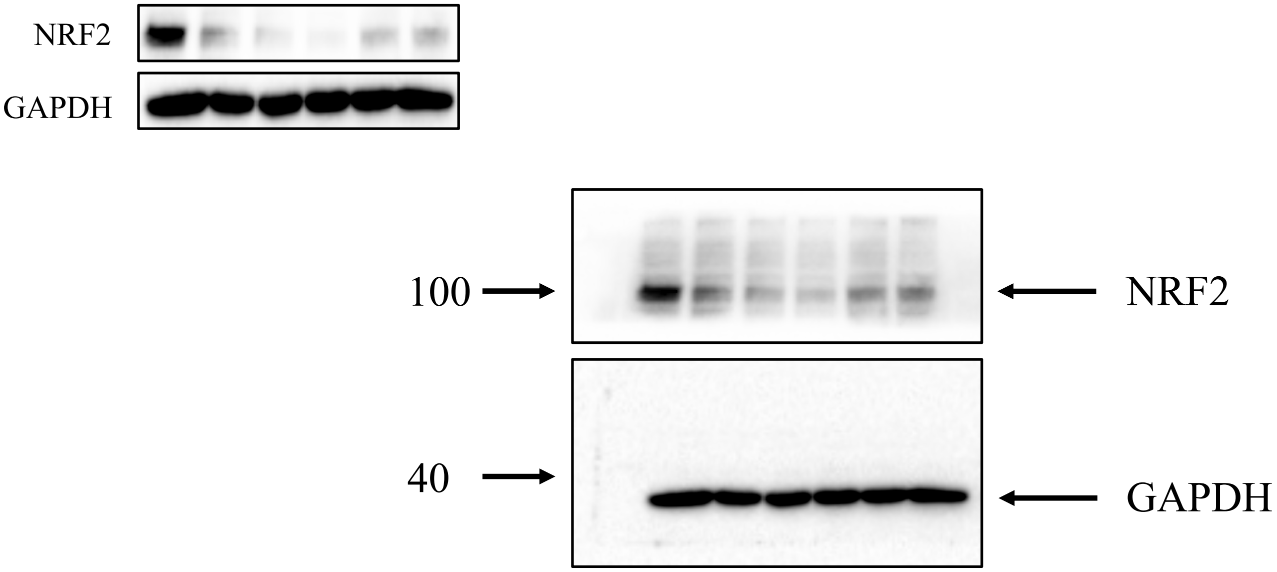

B

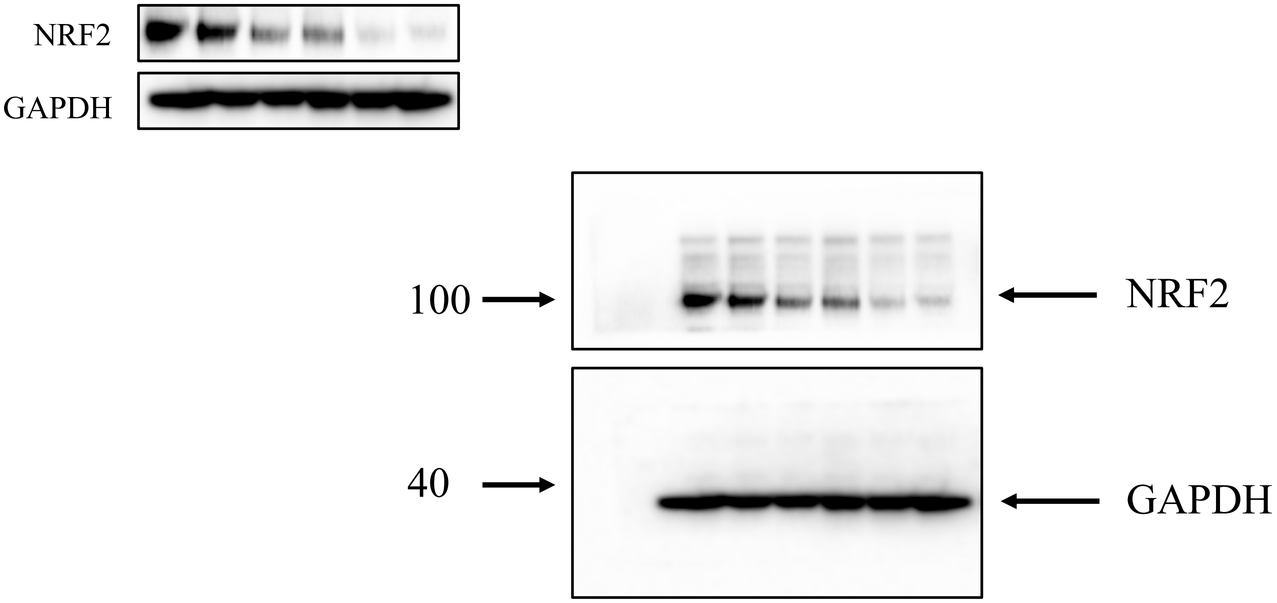

Figure S2

A

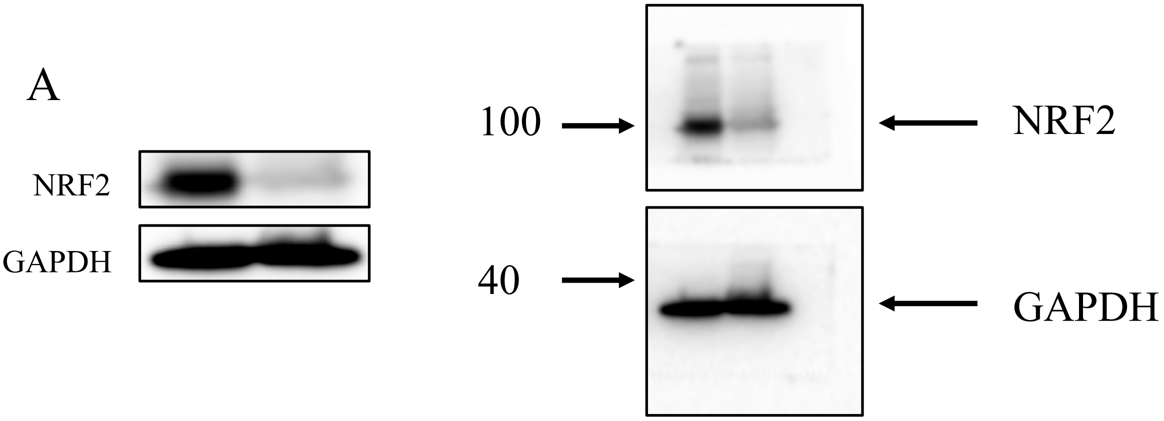

C

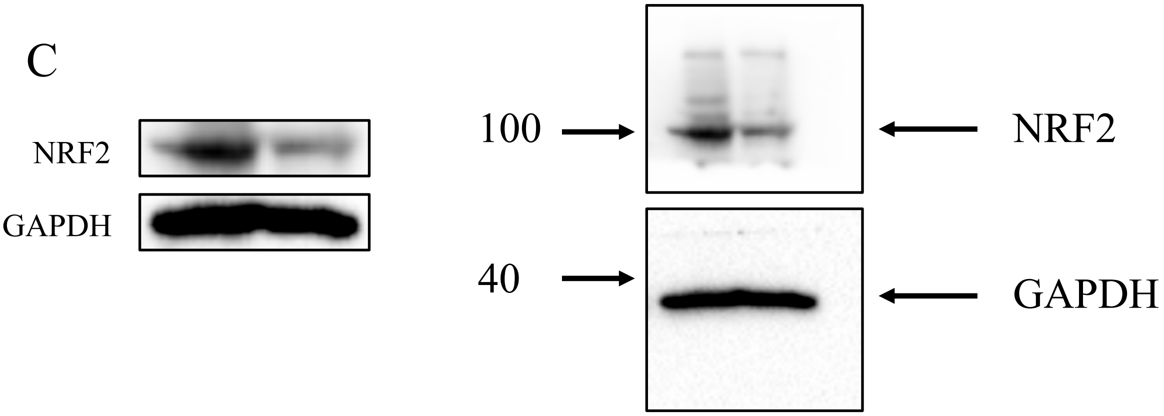

Figure S3

A

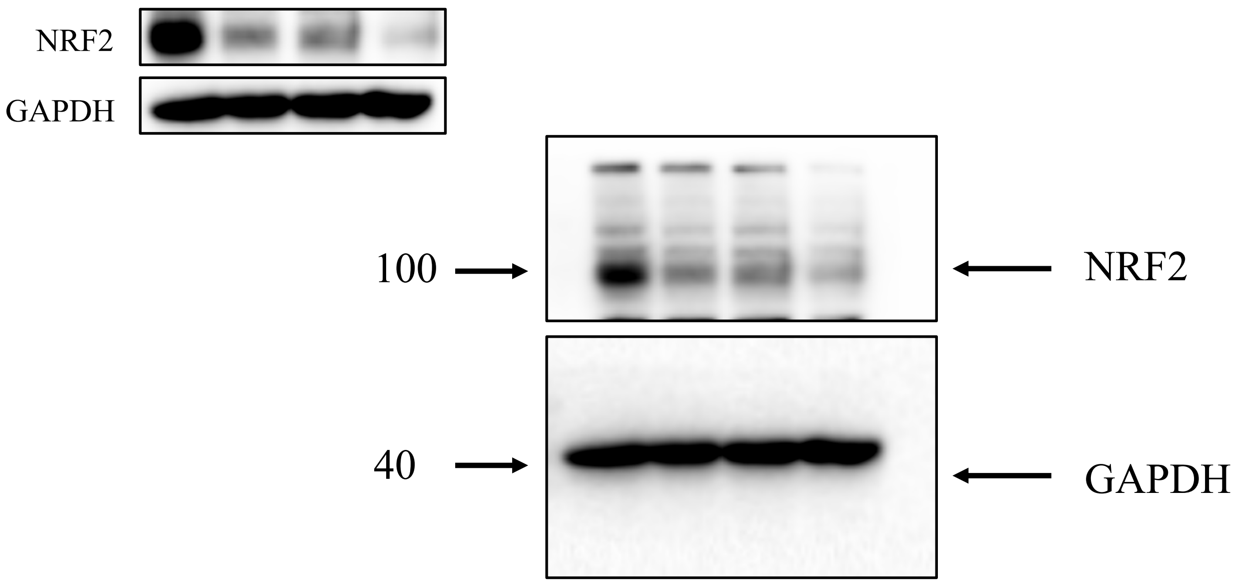

Supplement: Supplementary file 2 — Original Western Blots [file 41420_2025_2657_MOESM2_ESM.pdf]
